# Chronic arsenic exposure of ovarian surface and fallopian tube cultures induces giant and/or multinucleated cells with phagocytosis-like properties and an inflammatory phenotype^☆^

**DOI:** 10.1016/j.taap.2025.117394

**Published:** 2025-05-12

**Authors:** Cristina M. Andrade-Feraud, Arlet M. Acanda de la Rocha, Noah E. Berlow, Santiago Duque, Alexander Velazco, Diego Castillo, Baylee Holcomb, Ebony R. Coats, Yasmin R. Ghurani, Catherine M. Lucey, Brandon Pearson, Tomás R. Guilarte, Diana J. Azzam

**Affiliations:** a Department of Environmental Health Sciences, Robert Stempel College of Public Health & Social Work, Florida International University, Miami, FL, United States of America; b First Ascent Biomedical, Inc., United States of America; c Department of Environmental Health Sciences, Mailman School of Public Health, Columbia University, New York, United States of America; d Environmental and Molecular Toxicology, Oregon State University, OR, United States of America

**Keywords:** Arsenic, Chronic exposure, Ovary, Human primary cultures, Giant cells, Multinucleated cells, Chronic inflammation, DNA damage, NF-kB pathway, Carcinogenesis

## Abstract

Chronic exposure to arsenic, a toxic metalloid frequently found in groundwater and food, represents a significant environmental health risk and has been implicated in the etiology of several cancers, including ovarian cancer. However, the precise pathways through which arsenic exerts its toxic impact on the ovary are not fully understood. This study investigates the impact of chronic arsenic exposure at environmentally relevant concentrations (75 ppb or μg/L) on primary human ovarian surface (OCE1) and fallopian tube (FNE1) cultures derived from the same donor. These heterogeneous cultures provide a unique, human-relevant platform to investigate how chronic arsenic exposure influences distinct cell types within a shared microenvironment. Prolonged arsenic exposure induced significant cytotoxicity and promoted the formation of giant and/or multinucleated cells in both cultures. These cells exhibited phagocytosis-like properties, actively engulfing apoptotic debris. Transcriptomic analyses and pathway enrichment revealed robust activation of pro-inflammatory signaling, notably the canonical NF-κB pathway. This was marked by nuclear translocation of the NF-κB p65 subunit and elevated expression and secretion of pro-inflammatory cytokines, including TNFα, IL-6, and IL-8, driving a sustained inflammatory response. Moreover, arsenic-exposed cells displayed persistent DNA damage, as indicated by increased γ-H2AX foci, accompanied by nuclear structural alterations and elevated expression of cancer stem cell markers, including OCT2, CD133, and ALDH1. These findings suggest that arsenic-induced inflammation and genomic instability converge to promote a tumor-supportive microenvironment, highlighting the potential role of chronic arsenic exposure in ovarian carcinogenesis, particularly in the context of inflammation-driven carcinogenesis.

## 1. Introduction

Arsenic is a metalloid element ubiquitously present inthe environment, occurring in both organic and inorganic forms. Inorganic arsenic, commonly found in groundwater and various types of foods, is toxic and is a major source of human exposure (Humans et al., 2004). Arsenic-contaminated drinking water remains one of the most pressing global public health challenges (Naujokas et al., 2013). In the United States alone, an estimated 2.1 million individuals across more than 25 states are exposed to elevated arsenic concentrations in drinking water (Levin et al., 2024; Ayotte et al., 2017), exceeding the current regulatory limit of 10 parts per billion (ppb) (>10 μg/L) as set by the World Health Organization and the US Environmental Protection Agency (US EPA) (Ayotte et al., 2017; Subcommittee, 1999). In some regions of the United States, the level of arsenic in groundwater can be higher than 1500 ppb (μg/L) (Camacho et al., 2011). This widespread contamination has been documented in groundwater supplies across 108 countries, affecting approximately 40 million people globally (Rahman et al., 2009; Sen and Biswas, 2013; Wang and Wai, 2004). In addition to contaminated water, human exposure to arsenic occurs through inhalation of airborne particles, dermal absorption, and the consumption of arsenic-rich foods such as rice and rice-based products, which are particularly efficient at accumulating arsenic from soil and irrigation water (Chung et al., 2014).

Long-term exposure to arsenic can induce various cancers, including lung, bladder, skin, liver, prostate, and kidney cancer (Humans et al., 2004; Hong et al., 2014). There has also been growing evidence that low-level arsenic exposure, even at concentrations lower than the current drinking water regulatory level of 10 ppb (μg/L), still increases the risk of these types of cancers (Hasan et al., 2024; Roh et al., 2017; Mink et al., 2008). Alarmingly, a recent 2024 study detected trace amounts of arsenic, among other toxic metals, in multiple brands of tampons, raising the possibility that arsenic could cross the vaginal epithelium and enter systemic circulation (Shearston et al., 2024). The concern is increased by the ovary’s demonstrated vulnerability to arsenic’s toxic effects, as evidenced by animal studies showing transplacental carcinogenesis and the development of ovarian tumors in offspring exposed to arsenic. This was demonstrated when mice exposed during pregnancy to sodium arsenite (NaAsO_2_), a form of inorganic arsenic, at concentrations of 42.5 and 85 parts per million (ppm) in drinking water during pregnancy exhibited a dose-dependent increase in ovarian tumor development in female offspring (Waalkes et al., 2003; Tokar et al., 2011; Waalkes et al., 2004). This represents a previously underexplored route of exposure for women, with potentially significant implications for the initiation and progression of ovarian cancer, one of the deadliest cancers affecting women (Amin et al., 2019). Due to a lack of screening methods and nonspecific symptoms, over 70 % of ovarian cancers are diagnosed at stage III/IV when the tumors have already disseminated throughout the peritoneal cavity (Chornokur et al., 2013). Patients at this stage have a five-year survival rate of less than 30 %, a statistic that has not markedly improved over the past three decades (Cress et al., 2015). Although limited epidemiological studies have suggested a positive correlation between arsenic exposure and ovarian cancer (Amin et al., 2019; Cress et al., 2015), the precise pathways through which arsenic exerts its toxic impact on the ovary are not yet fully understood. Addressing this critical gap in knowledge is essential to improving early detection and therapeutic outcomes for this devastating disease.

Chronic arsenic exposure has been shown to disrupt DNA repair pathways, induce oxidative stress, alter immune function, and create a pro-inflammatory microenvironment conducive to tumor growth in multiple cell models (Giles and Mann, 2022; Jomova et al., 2011; Tam et al., 2020). Notably, arsenic exposure has also been shown to transform human epithelial stem/progenitor cells into a cancer stem-like phenotype (Tokar et al., 2010a; Tokar et al., 2010b). However, there is limited evidence regarding the direct effects of chronic low-level arsenic exposure on human ovarian cultures, which are primary cultures and distinct from established ovarian epithelial cell lines. Primary cultures are heterogeneous and more closely resemble the *in vivo* environment (Merritt et al., 2013; Ince et al., 2015). Additionally, it is unclear whether the cell-of-origin may impact transformation. Therefore, this study investigated the effects of chronic exposure to 75 ppb (μg/L) of arsenic in normal human cultures derived from the surface of the ovary (OCE1 cells) and the fallopian tubes (FNE1 cells), obtained from the same donor (Merritt et al., 2013).

## 2. Materials & methods

### 2.1. Primary ovarian surface and fallopian tube cultures and arsenic exposure

Ovarian surface (OCE1) and fallopian tube (FNE1) cultures were developed by the lab of Dr. Tan Ince and obtained from Culture Collections, UK. Matched sets of cultures from the ovarian surface and fallopian tube were harvested from a postmenopausal female donor during surgical treatment for benign gynecological conditions. These cultures were maintained in Fallopian Tube and Ovary Media Ince (FOMI) media (USBiological Life Sciences, Cat# 506388) and supplemented with 0.5–1 % FBS (Thermo Fisher Scientific Inc., Waltham, MA, Catalog# A56708–01). Immortalization was carried out by transfection of the pmig-GFP-hTERT vector to produce normal immortalized cultures (OCE1 and FNE1). Both OCE1 and FNE1 cultures maintain their phenotype during long-term cultures and retain histotype-specific features of their tissue of origin, similarity was 90–95 % when comparing the samples genomic loss-of-heterozygosity, as confirmed by Reverse-Phase Protein Array (RPPA) studies (Merritt et al., 2013; Ince et al., 2015). Cells were grown in Primaria-treated flasks (Corning, Catalog# 353810) at 37°C in a humidified incubator with 5 % CO_2._ Media was changed every three to four days.

For chronic arsenic exposure experiments, OCE1 and FNE1 cultures were exposed to sodium arsenite (NaAsO_2_) diluted in water at 1 μM for over six weeks, with vehicle-treated cells serving as controls. Cell proliferation was monitored in real-time using the Incucyte ZOOM Live Cell Analysis System, with media changed every three to four days and weekly cell passaging, as noted below. In the washout experiment, arsenic exposure was removed after three weeks, and the cells were cultured in arsenic-free media for up to three more weeks to observe any changes post-exposure. Additionally, cultures were treated with other metals, including 1 μM cadmium chloride and 1 μM lead (III) acetate diluted in water, to assess the effects of chronic exposure to various toxic metals. The effects on cell viability and proliferation were similarly monitored, with exposures maintained for up to six weeks.

### 2.2. Cell viability assay

OCE1 and FNE1 cell viability was measured using trypan blue (Fisher Scientific, Cat#15–250-061) staining in a 1:1 ratio. Cells were counted using an automated cell counter (Invitrogen, Countess Cell Counter). Cell viability was expressed as the percentage of live cells, and arsenic-exposed cells were compared to the control over time in at least six independent experiments for each time point and cell type.

### 2.3. Incucyte live cell imaging system

After two weeks of chronic arsenic exposure, vehicle and arsenic-exposed cultures were seeded at 6000 cells/well in a 24-well plate with their appropriately treated media for continued exposure in duplicates. Real-time cell proliferation was measured using Incucyte ZOOM Live Cell Analysis System (2016B software version), taking nine different images of each well every 30 min at 10× magnification. Cell culture media was changed every three days and cells were split every week to maintain a density of 6000 cells/well. Cell proliferation was assessed for a total of six weeks of chronic exposure. Cell proliferation was calculated by the total live cell area per well over time in at least 3 independent experiments.

### 2.4. Assessment of giant cells (GCs) and multinucleated cells (MNCs)

Cells were plated at 10,000 cells/well in 24-well plates after three weeks and six weeks of chronic arsenic exposure. Cells were subsequently fixed with 4 % PFA (paraformaldehyde) and mounted using Invitrogen ProLong Gold Antifade mounting with DAPI nuclear stain solution (Thermo Fisher Scientific, Cat# P36935). *Z*-stack images were taken using confocal microscopy (Olympus, Fluoview, FV10i) and analyzed using ImageJ (http://imagej.nih.gov) to calculate the size of the nuclear area and perimeter and to quantify the number of giant and multinucleated cells. GCs were defined as any cell with a nucleus at least three times the standard deviation of the mean area or perimeter of the vehicle nuclei, and MNCs as any cell with more than one nucleus. For cells classified as both multinucleated and giant, the total nuclear area and perimeter were calculated by summing the measurements of all nuclei within the cell. Statistical analysis was performed on the area, perimeter, and percentage of GCs and MNCs in at least 4 independent experiments.

### 2.5. Quantitative real-time RT-PCR

Total RNA was extracted from the arsenic-exposed cells and vehicle OCE1 and FNE1 cells at the indicated time points using RNeasy Mini Kit (Qiagen, USA), per the manufacturer’s instructions. RNA was reverse transcribed using High-Capacity cDNA Reverse Transcription Kit (Thermo Fisher, Cat# 4368813). Real time-qPCR was performed using TaqMan Fast Advanced Master Mix (Thermo Fisher, Cat# 4444557), per the manufacturer’s instructions to detect the pro-inflammatory genes (*IL1α*, *IL1β*, *IL6*, *IL8*, *IL10*, *TNFα*). All results were normalized to GAPDH and the quantitative method of relative mRNA expression used was 2^−ΔΔCt^ in at least 4 independent experiments (Livak and Schmittgen, 2001).

### 2.6. Cellular immunofluorescence

Cells (10,000 cells/well) were plated on coverslips in 24-well plates. Cells were subsequently fixed at the indicated time points (three weeks and six weeks of chronic arsenic exposure) with 4 % paraformaldehyde at room temperature for 10 min. Following fixation, cells were permeabilized and blocked using a solution containing 5 % bovine albumin serum (BSA), 0.2 % Tween-20, and 10 % Triton X-100 in PBS for 45 min. Cells were incubated overnight at 4 °C with the following primary antibodies in a humidified chamber: NF-κB p65 (1:1000; cat. no. 8242; Cell Signaling Technology, Inc.), γ-H2AX (1:1000; cat. no. 9718; Cell Signaling Technology, Inc.). The following day, cells were incubated for 30 min at room temperature in a humidified chamber with Alexa Fluor 555 dye-conjugated secondary antibody (1: 250, cat. no.4413S; Cell Signaling Technology, Inc.). After each incubation, cells were washed with 0.2 % Tween-20 in Hanks’ Balanced Salt Solution (HBSS) three times for 5 min on a shaker. Coverslips were subsequently mounted with 5 μL of ProLong^™^ Gold Antifade Mountant with DAPI (cat. no. P36935; ThermoFisherScientific). Fluorescent images were acquired using an Olympus Fluoview FV10i confocal laser microscope. The mean immunofluorescence intensity and the proportion of positive cells were quantified using ImageJ software (version 1.48v) in at least three independent experiments. *Z*-stack imaging was employed to distinguish nuclear and cytoplasmic signals. Nuclear signal intensity was compared to cytosolic signal intensity by calculating cytosolic intensity using the formula:

CytosolicSignalIntensity=IntegratedDensity(Cytosol)−IntegratedDensity(Nucleus)/CytosolArea−NucleusArea

### 2.7. Meso-Scale Discovery (MSD)

Protein expression of inflammatory cytokines (IL-10, IL-1α, IL-1β, IL-8, IL-6, and TNFα) were quantified using a multi-spot assay system V-Plex kit (Meso Scale Discovery, Rockville, MD, USA) as per the manufacturer’s instructions using the cytokines at different dilutions: IL-1α [undiluted], IL-10, IL-1β, IL-6, and TNFα [1:10], and IL8 [1:8000]. Cytokine levels were analyzed in media from arsenic-exposed and vehicle-treated cells using a Meso Quick Plex SQ 120 nm plate reader (Meso Scale Discovery) in at least five independent experiments.

### 2.8. RNA Seq analysis

At the indicated time points (three weeks and six weeks of chronic arsenic exposure), total RNA was extracted from arsenic-exposed or vehicle-exposed FNE1 and OCE1 cells using the RNeasy Mini Kit (Qiagen, USA), per the manufacturer’s instructions. RNA purity and concentration were measured using a Nanodrop One spectrophotometer (Thermo Scientific, USA). Quality control, library preparation, and physical sequencing of isolated RNA were performed by Novogene using the Illumina NovaSeq PE150 platform. The resulting raw RNA sequencing data were analyzed using a previously established gene expression analysis pipeline based on best practices (Acanda De La Rocha et al., 2024; Berlow et al., 2019). In brief, FASTQ sequencing files underwent QC filtering *via* SOAPnuke v2.1.8 (Chen et al., 2018), GRCh38 human reference transcriptome alignment using STAR v2.7.10b (Dobin et al., 2013), and gene expression quantification using RSEM v1.3.3 (Li and Dewey, 2011).

Differential gene expression between time and exposure groups was performed using the edgeR 3.42.4 package in R 4.3.0, with differentially expressed genes defined as those with 1) count per million >1 for at least two samples, 2) adjusted *p*-value <0.05 following Benjamini-Hochberg multiple comparison corrections, and 3) log2 (fold change) less than − 0.5 or greater than 0.5. Differentially expressed genes were compared against databases of published annotation signatures using the online DAVID software tool (Database for Annotation, Visualization, and Integrated Discovery, https://david.ncifcrf.gov/). Specifically, DAVID analysis was performed on differentially expressed gene sets in arsenic-exposed FNE1 and OCE1 cells at week six as compared to vehicle-treated cells (five independent experiments for FNE1 cells and three independent experiments for OCE1 cells). Differentially expressed common genes were identified among OCE1 and FNE1 cells at week six of exposure using Venn diagram analysis using www.bioinformatics.com/gvenn.

OCE1 and FNE1 cultures were characterized using quanTIseq (Finotello et al., 2019), a commonly used bulk RNA-seq deconvolution tool (five independent experiments for OCE1 and FNE1 cultures). Differential cell populations with cancer stem cell markers present in the arsenic-exposed cohort were quantified using MCP Counter, using a list of established cancer stem cell gene markers relevant to ovarian, cervical, and endometrial cancer stem cells (Chu et al., 2024) (five independent experiments for FNE1 week three and week six, five independent experiments OCE1 week three of exposure, and three independent experiments for OCE1 week six of exposure).

### 2.9. Statistical analysis

All statistical analyses of these studies were performed using the statistical program GraphPad Prism version 7. Data were presented as mean ± standard error from at least three independent experiments. The normal distribution was assessed with the Shapiro-Wilk test. The differences between the two groups were analyzed using a two-tailed, unpaired Student’s *t*-test, and a one-way analysis of variance was used to compare the means amount of three or more groups, where *p* < 0.05 was considered statistically significant. Multiple exposure groups were compared with the control group using Tukey. Each experiment was repeated at least three times to provide sufficient statistical power to detect significant differences between the groups.

For DAVID analysis of differentially expressed genes, Bonferroni multiple comparison correction was used to adjust *p*-values for multiple hypothesis testing, performed *via* the online DAVID analysis tool. Comparison of immune cell populations was performed *via* multivariate ANOVA in GraphPad Prism 10.4.1, with Sidak’s correction for post-hoc comparisons. The comparison of macrophage population counts was performed using Student’s *t*-test with Welch’s correction in GraphPad Prism 10.4.1. Comparison of cancer stem cell counts was performed using ANOVA with Tukey’s multiple comparison correction in GraphPad Prism 10.4.1.

## 3. Results

### 3.1. Chronic arsenic exposure elicits cytotoxic effects and promotes the formation of giant and/or multinucleated cells in human ovarian surface and fallopian tube cultures

**Fig. 1.**
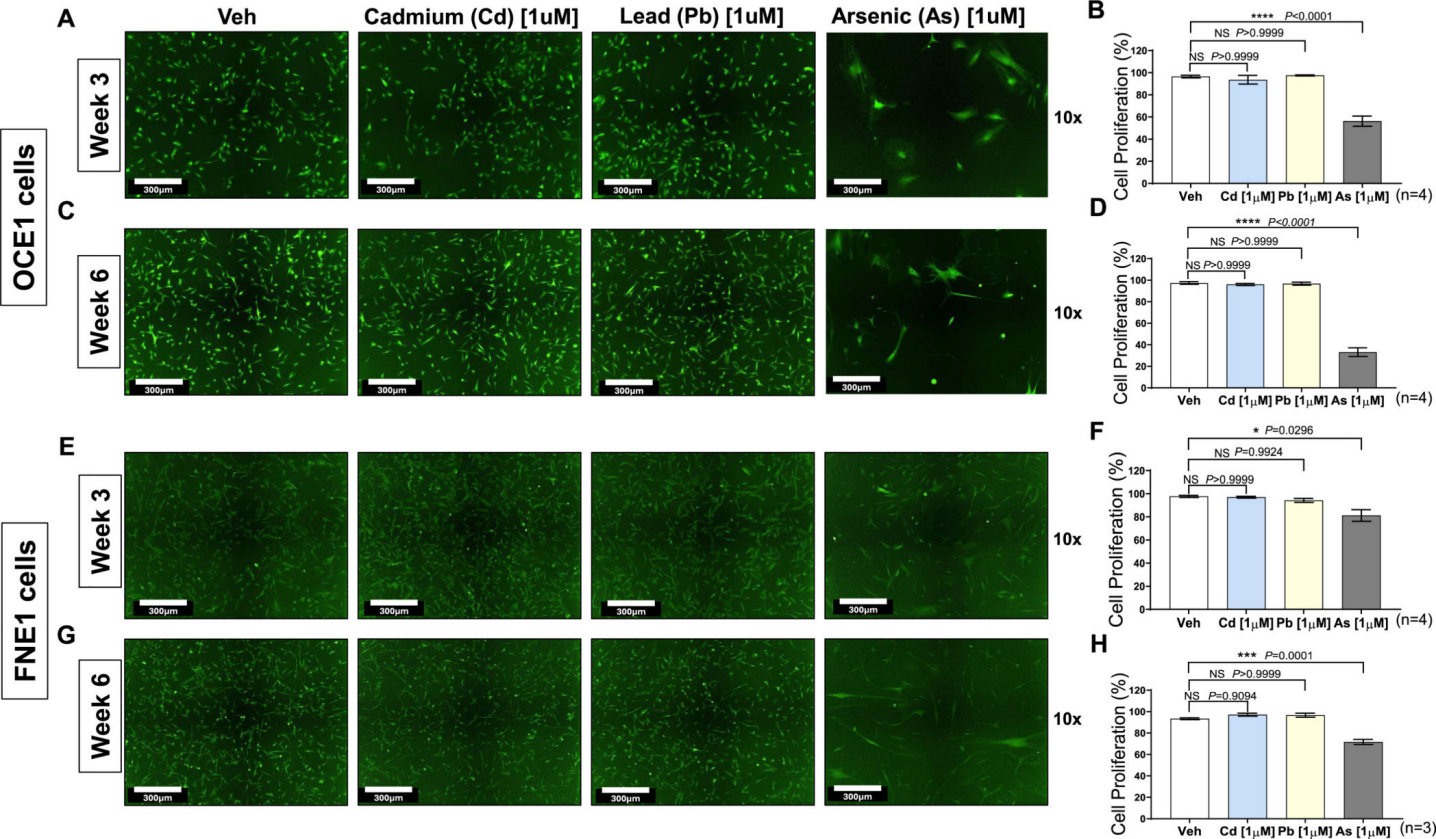
Time-Dependent Arsenic-Specific Effect on Cell Proliferation in OCE1 and FNE1 Cells. OCE1 and FNE1 cells were chronically exposed to 1 μM of NaAsO_2_ (As), cadmium chloride (Liu et al., 2013), lead acetate (Pb), or vehicle (Veh) for 3 and 6 weeks, and monitored using the Incucyte Live Cell Imaging System. Representative live-cell images of OCE1 cells after 3 weeks (A) or 6 weeks (C) of exposure and FNE1 cells after 3 weeks (E) or 6 weeks (G) of exposure. Quantification of cellular proliferation in OCE1 cells after 3 weeks (B) or 6 weeks (D) of exposure and FNE1 cells after 3 weeks (F) or 6 weeks (H) of exposure, as determined by the total live cell area per well over time, using the Incucyte ZOOM Live Cell Analysis System. Data are presented as mean ± SEM of at least three independent experiments (*n* = 3–4). Statistical significance was determined using a two-tailed unpaired *t*-test (*p < 0.05, **p < 0.01, ***p < 0.001, ****p < 0.0001). Scale bars represent 300 μm. Images were captured at 10× magnification.

To evaluate the specific effects of chronic arsenic exposure on human ovarian cells, matched primary cultures derived from the ovarian surface (OCE1) and fallopian tube (FNE1) of the same donor were exposed to [1 μM] arsenic, cadmium chloride, lead acetate or vehicle control for up to six weeks. These primary cultures are heterogeneous, comprising mixed cell populations as confirmed by quanTIseq, a bulk RNA sequencing deconvolution tool (Supplementary Fig. 1). The chosen arsenic concentration is equivalent to approximately 75 ppb (μg/L), which is significantly lower than arsenic levels documented in countries such as Spain, Mexico, Japan, India, China, Canada, Chile, Bangladesh, Bolivia, and Argentina, where levels frequently exceed 300 ppb (μg/L) (Humans et al., 2004). Among the tested toxic metals, arsenic uniquely induced a significant, time-dependent decrease in cell proliferation and increase in cytotoxicity in both OCE1 and FNE1 cultures, compared to cadmium chloride and lead acetate, and vehicle-treated controls. These effects were evident after three weeks of exposure (Fig. 1A–B for OCE1; Fig. 1E–F for FNE1) and 6 weeks of exposure (Fig. 1C–D for OCE1; Fig. 1G–H for FNE1). Interestingly, chronic arsenic exposure elicited distinct morphological changes in the surviving OCE1 and FNE1 cells. As shown in Fig. 1, chronic arsenic exposure led to a marked enrichment in the formation of giant cells, suggesting potential mechanisms of cellular stress and adaptation in response to prolonged arsenic exposure. The formation of giant cells was exclusively observed following arsenic exposure, a morphological alteration not observed in cadmium chloride or lead acetate-treated cells. Consistent with these findings, cell viability was significantly reduced in arsenic-exposed OCE1 and FNE1 cultures compared to controls, with effects observed at both time points (Supplementary Fig. 2A–D for OCE1; Supplementary Fig. 2E–H for FNE1).

To assess the potential reversibility of cellular effects caused by chronic arsenic exposure, OCE1 and FNE1 cultures were exposed to 1 μM arsenic for three weeks, followed by a three-week washout period in arsenic-free media to evaluate recovery. As shown in Supplementary Fig. 3, chronic arsenic exposure resulted in inhibition of cell proliferation compared to vehicle-treated controls. However, upon arsenic removal from the culture medium, surviving cells, including giant cells, exhibited continued proliferation. A statistically significant increase in FNE1 cells was observed at three weeks post-washout compared to one-week post-washout (Supplementary Fig. 3C–D). Although a similar trend in proliferation was observed in OCE1 cells, the difference was not statistically significant (Supplementary Fig. 3A–B). Notably, the arsenic-induced giant cells remained viable in culture even three weeks after arsenic removal, indicating their sustained proliferative capacity.

**Fig. 2.**
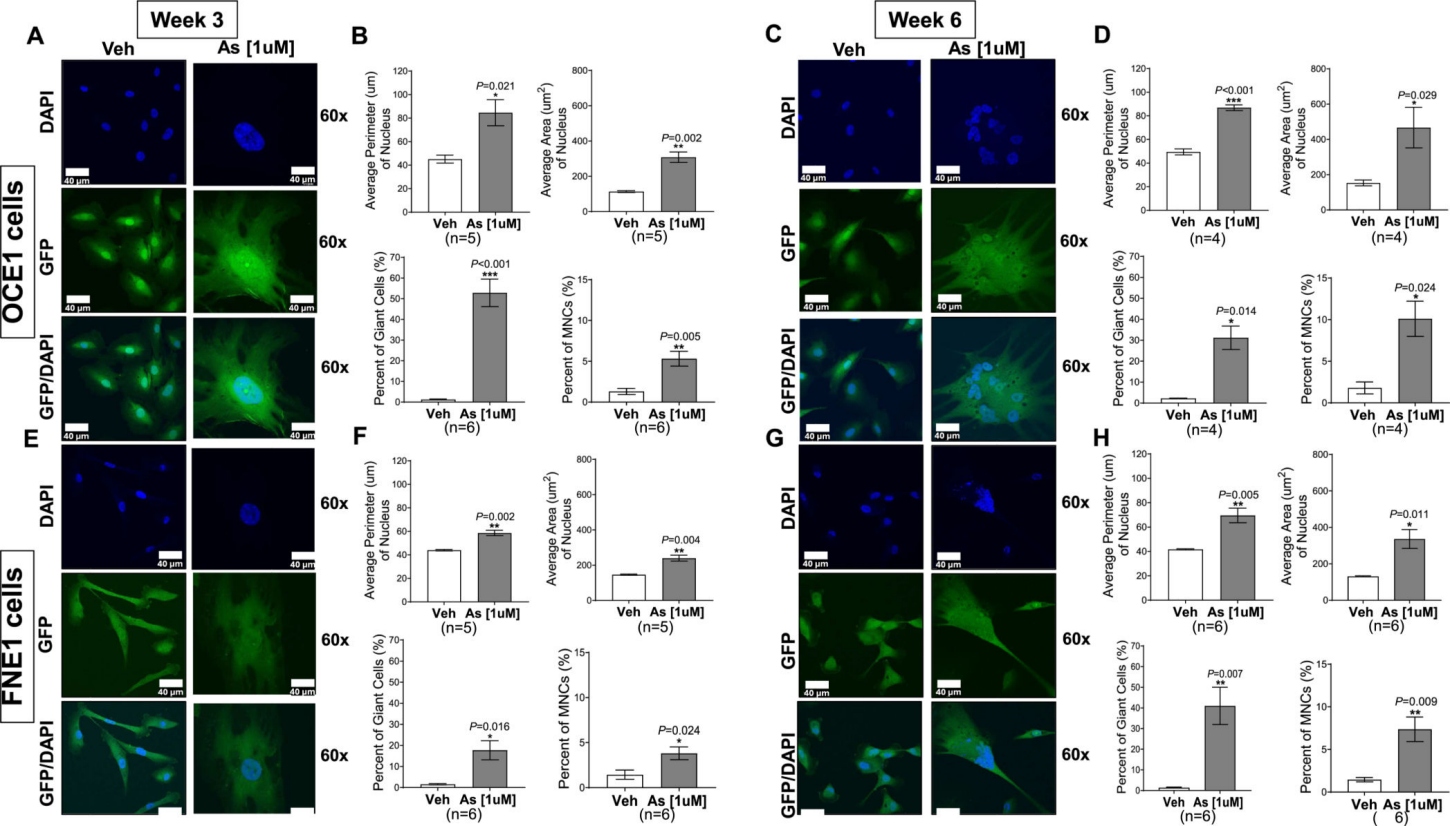
Chronic Arsenic Exposure Induces the Formation of Giant Cells (GCs) and Multinucleated Cells (MNCs) in OCE1 and FNE1 Cells Over Time. OCE1 and FNE1 cells were exposed to 1 μM NaAsO_2_ (As) or vehicle (Veh) for 3 and 6 weeks. At each time point, cells were fixed with 4 % PFA and stained with DAPI to visualize nuclei (blue). Representative confocal images of OCE1 cells at 60× magnification after 3 weeks (A) or 6 weeks (B) of exposure to As or Veh, and of FNE cells after 3 weeks (E) or 6 weeks (G) of exposure. Images show DAPI in blue, GFP in green and the merged channel. Scale bar: 40 μm. Quantification of average perimeter (μm) of nucleus, the average area (μm^2^) of the nucleus, the percentage (%) of giant cells, and the % of multinucleated cells (MNCs) in As-exposed OCE1 cells compared to Veh at 3 weeks (B) or 6 weeks (D) and in FNE1 cells at 3 weeks (F) or 6 weeks (H) after exposure All quantitative analyses were performed using data from 4 to 6 independent experiments, analyzing a total of 10,000 cells. Data were statistically evaluated using a two-tailed unpaired *t*-test to identify significant differences between arsenic-treated and vehicle control groups (*p < 0.05, **p < 0.01, ***p < 0.001).

To examine the morphological effects of chronic arsenic exposure, nuclear size—measured as nuclear area and perimeter—was analyzed in OCE1 and FNE1 cultures at three-week and six-week time points following exposure to 1 μM arsenic. Quantitative analysis revealed a significant increase in nuclear area and perimeter in both OCE1 (Fig. 2A–D) and FNE1 cells (Fig. 2E–H) after three or six weeks of arsenite exposure, respectively, compared to vehicle-treated controls. Confocal microscopy further confirmed a significant increase in the number of giant cells (GCs) and multinucleated cells (MNCs) in both OCE1 and FNE1 populations after chronic arsenic exposure as compared to vehicle-treated controls. GCs were defined as cells with nuclei at least three times the standard deviation of the mean nuclear area or perimeter in vehicle-treated cells, while MNCs were defined as cells containing more than one nucleus (Acanda De La Rocha et al., 2024). For cells classified as both multinucleated and giant, the total nuclear area and perimeter were calculated by summing the measurements of all nuclei within the cell. The majority of the cells following arsenic exposure are GCs with a subset of those (approximately 10–15 %) being multinucleated - a proportion that increased over time (Fig. 2). These results indicate that chronic arsenic exposure induces substantial changes in nuclear morphology and promotes the formation of GCs and/or MNCs.

To further investigate the formation and enrichment of GCs and/or MNCs under chronic arsenic exposure, real-time imaging was conducted to monitor cellular dynamics over time. As shown in Supplementary Videos 1–6, a time-dependent increase in cell death was observed in both OCE1 and FNE1 cells exposed to 1 μM arsenic compared to vehicle-treated controls, indicated by Cytotox Red dye uptake, which identifies compromised cell membranes. Remarkably, live-cell imaging uncovered a previously unreported phenomenon; that is, surviving arsenic-exposed OCE1 and FNE1 GCs/MNCs actively engulfed apoptotic remnants from neighboring dying cells over 7 days (Supplementary Videos 1–6). This cellular behavior, resembling phagocytosis, suggests that the surviving cells may acquire additional resources by engulfing apoptotic debris, such as nutrients, or receive survival-promoting signals. These adaptations likely enhance their resilience to chronic toxic stress, thereby facilitating the accumulation and enrichment of GCs and MNCs following arsenic exposure.

### 3.2. Chronic arsenic exposure induces indirect DNA damage in human ovarian surface and fallopian tube cultures

**Fig. 3.**
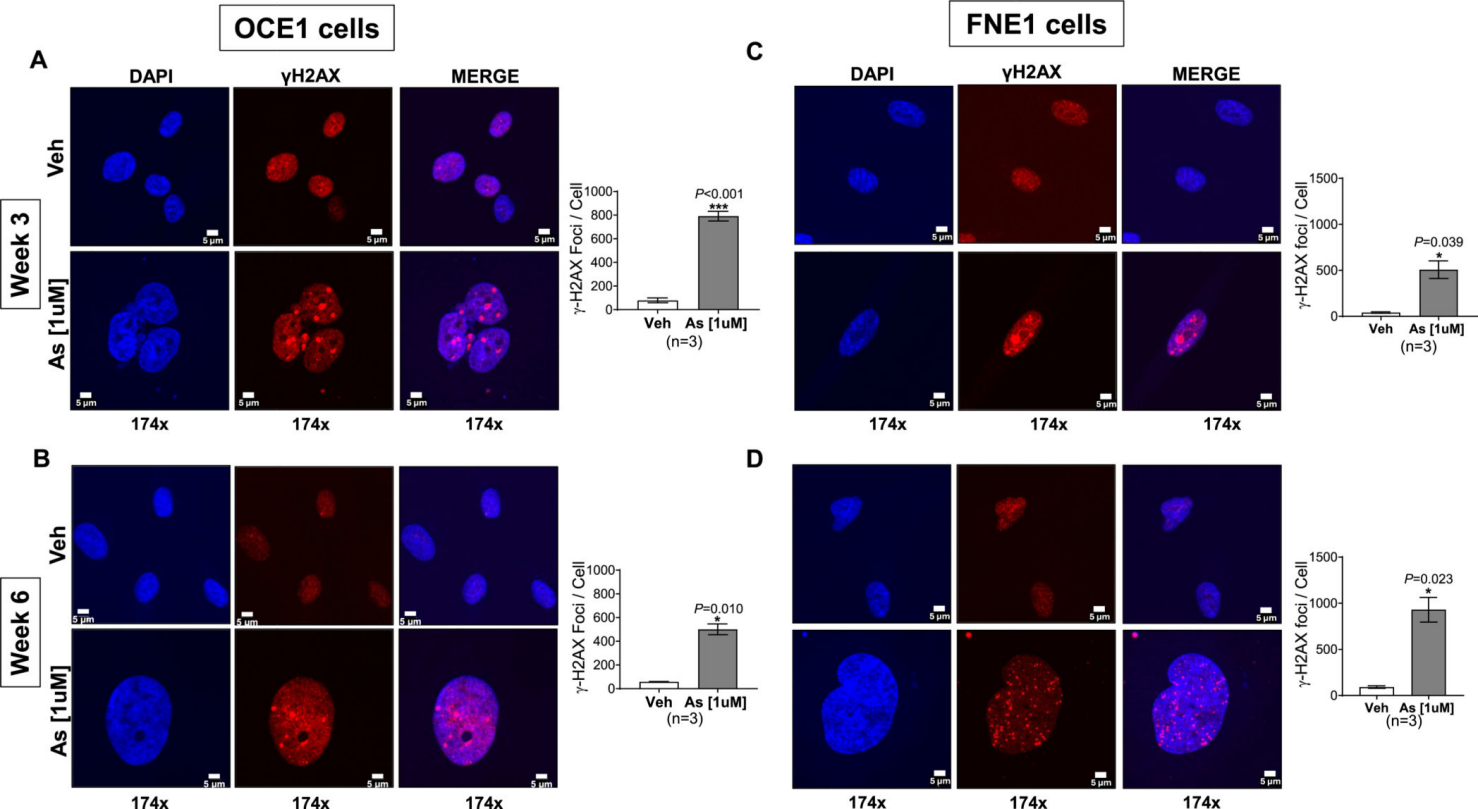
Chronic Arsenic Exposure Increases DNA damage by Accumulating γH2AX foci in OCE1 and FNE1 cells. OCE1 and FNE1 cells were exposed to 1 μM NaAsO_2_ (As) or vehicle (Veh) for 3 and 6 weeks. OCE1 Cells: Representative immunofluorescent images at 174× magnification of γH2AX (red) and DAPI (blue) in OCE1 cells after 3 weeks (A) and 6 weeks (B) of exposure, and in FNE1 cells after 3 weeks (C) or 6 weeks (D) of exposure (Scale bar: 5 μm). Bar graphs show the quantitative analysis of γH2AX foci per cell in As-exposed cells compared to Veh in both cell types at 3 and 6 weeks, respectively. Data are represented as mean ± SEM from 3 independent experiments (*n* = 3). Statistical significance was determined using a two-tailed unpaired *t*-test to identify significant differences between arsenic-treated and vehicle control groups (*p < 0.05, **p < 0.01, ***p < 0.001).

To elucidate the mechanisms contributing to multinucleated cell formation and genomic instability induced by chronic arsenic exposure, we evaluated the accumulation of γ-H2AX foci, a well-established marker of DNA double-strand breaks, in OCE1 and FNE1 cells following three and six weeks of 1 μM chronic arsenic exposure. As shown in Fig. 3, a significant increase in nuclear γ-H2AX foci was observed in both OCE1 (Fig. 3A–B) and FNE1 cultures (Fig. 3C–D) at both time points. In contrast, vehicle-treated controls exhibited minimal or undetectable γ-H2AX foci. These findings imply that DNA damage may be indirectly associated with nuclear remodeling, ultimately contributing to genomic instability and the development of a giant and/or multinucleated phenotype.

### 3.3. Activation of NF-kB pathway and pro-inflammatory response following chronic arsenic exposure in human ovarian surface and fallopian tube cultures

To identify molecular pathways altered by chronic arsenic exposure in ovarian surface and fallopian tube cultures, we analyzed gene expression profiles of OCE1 and FNE1 cultures following six weeks of exposure to 1 μM arsenic. Venn diagram analysis (Supplementary Fig. 4) identified 30 downregulated and 68 upregulated differentially expressed genes (DEGs) common to both cell types (Supplementary Table 1). Pathway enrichment analysis of the shared downregulated DEGs revealed significant suppression of cholesterol metabolism (Supplementary Fig. 4), indicating potential disruption of lipid homeostasis. In contrast, pathway enrichment analysis of the common upregulated DEGs highlighted significant activation of key inflammatory and immune-related pathways such as cytokine-receptor interaction, NF-κB signaling, and chemokine signaling pathways (Supplementary Fig. 4).

**Fig. 4.**
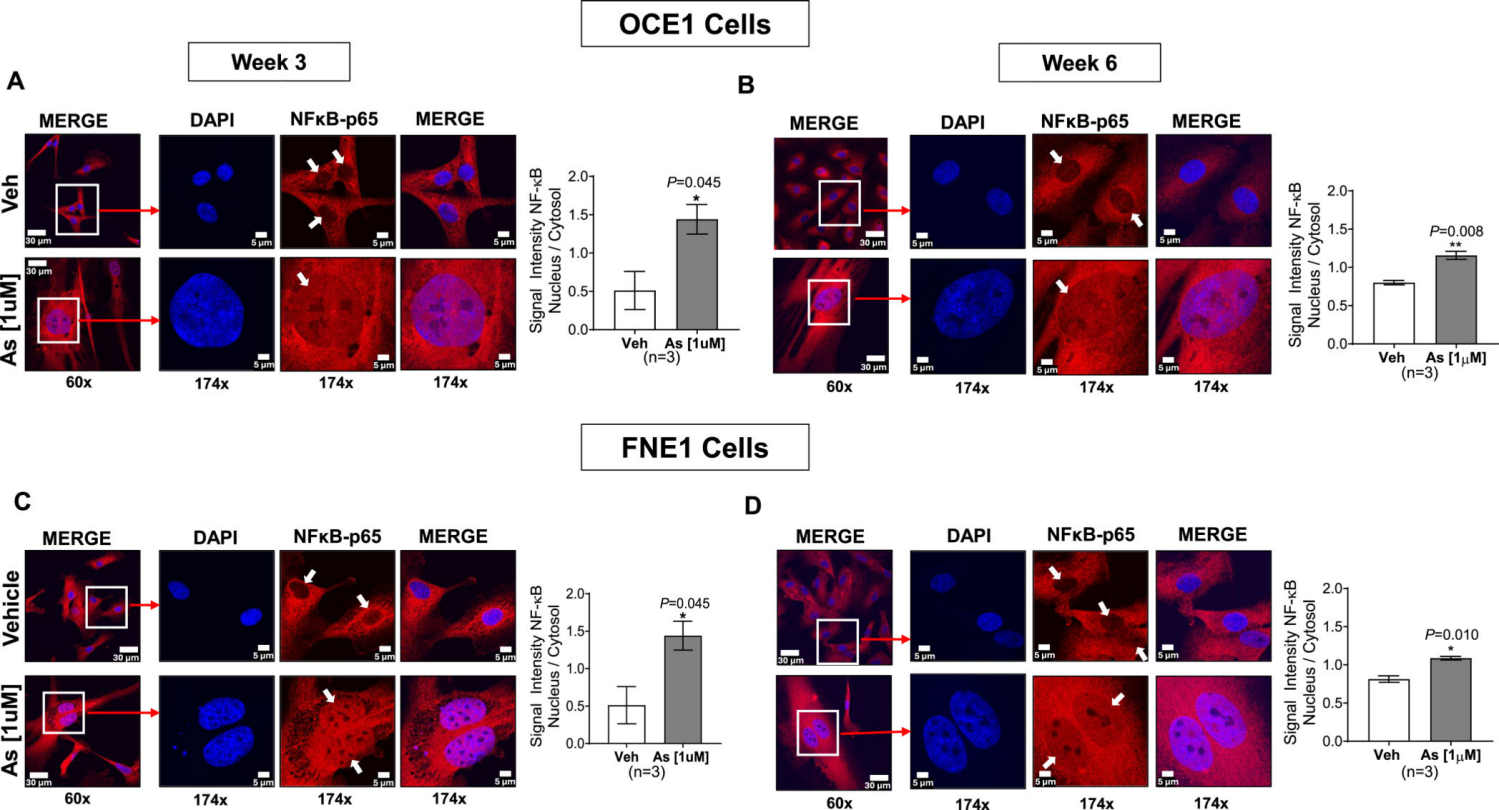
Arsenic Induces Nuclear Translocation of NF-ĸB p65 in OCE1 and FNE1 cells. OCE1 and FNE1 cells were exposed to 1 μM NaAsO_2_ (As) or vehicle (Veh) for 3 and 6 weeks, followed by immunofluorescence staining to detect NF-ĸB p65 (red) and nuclei (DAPI, blue). Representative immunofluorescent images of OCE1 cells after 3 weeks (A) or 6 weeks (B) of exposure and for FNE1 cells after 3 weeks (C) or 6 weeks (D) of exposure, were captured at 60× magnification (scale bar: 30 μm) and 174× magnification (scale bar: 5 μm). Bar graphs show quantitative analysis of the ratio of nuclear-to-cytosolic signal intensity ratios of NFκB-p65 in As-exposed cells compared to Veh. Data are represented as mean ± SEM from three independent experiments (n = 3). Statistical significance was determined using a two-tailed unpaired *t*-test (*p < 0.05, **p < 0.01, ***p < 0.001).

Given that RNA-seq analysis identified the canonical NF-κB signaling pathway as a key target, we investigated the intracellular localization of the NF-κB p65 subunit—a hallmark of pathway activation—after three and six weeks of chronic arsenic exposure. As shown in Fig. 4, arsenic exposure significantly increased nuclear translocation of the p65 subunit in both cultures at both time points (Fig. 4A–B for OCE1; Fig. 4C–D for FNE1), confirming activation of the canonical NF-κB pathway. This activation suggests a functional link between indirect DNA damage, genomic instability, and the induction of survival and stress response pathways in response to chronic arsenic exposure.

**Fig. 5.**
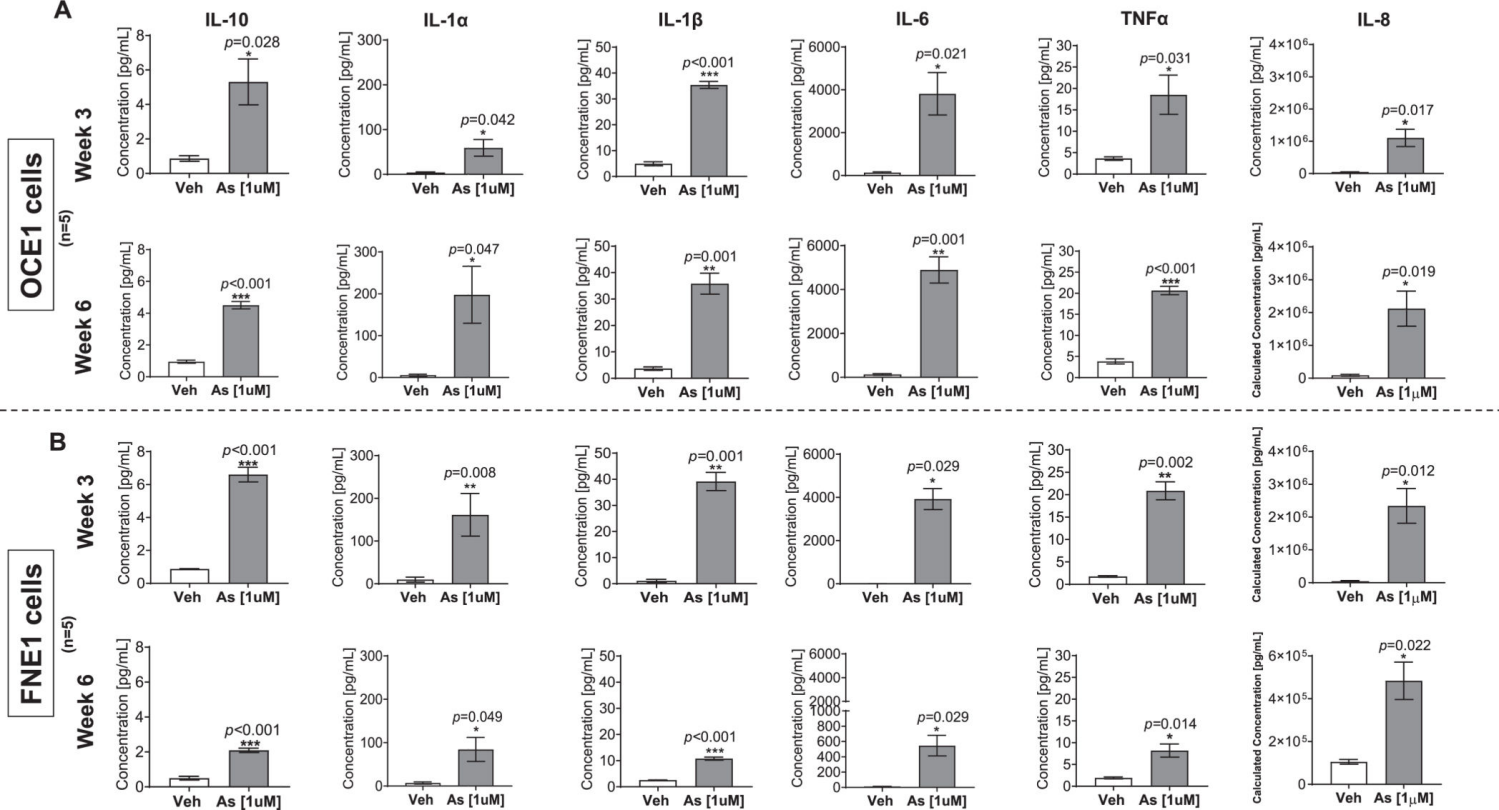
Chronic Arsenic Exposure Increases Pro-inflammatory Cytokines Secreted by OCE1 and FNE1 Cells. OCE1 and FNE1 cells were exposed to 1 μM NaAsO_2_ (As) or vehicle (Veh) for 3 and 6 weeks. The levels of secreted pro-inflammatory cytokines, including TNFα, IL-10, IL-1α, IL-1β, IL-6, and IL-8, were quantified in the culture supernatant of OCE1 cells after 3 weeks (A; upper panels) or 6 weeks (A; bottom panels) of exposure, and in FNE1 cells after 3 weeks (B; upper panels) or 6 weeks (B; bottom panels) of exposure using Meso-Scale Discovery (MSD) system. Cytokine concentrations were normalized to cell counts and are presented as mean protein levels (pg/mL) from 5 independent experiments (*n* = 5). Statistical analysis was performed using a two-tailed unpaired t-test to assess differences between groups (*p < 0.05, **p < 0.01, ***p < 0.001).

Chronic arsenic exposure also triggered a pronounced pro-inflammatory response in ovarian surface and fallopian tube cultures. RNA-seq analysis revealed consistent upregulation of key pro-inflammatory genes, including *IL1α*, *IL1β*, *IL6*, *IL8*, *IL10*, and *TNF-α*, contributing to the enrichment of chemokine signaling and inflammatory response pathways. Validation by RT-qPCR confirmed that the mRNA expression of these genes was significantly increased in OCE1 and FNE1 cells at both three- and six-week time points (Supplementary Fig. 6A–B). Protein levels of proinflammatory cytokines were also markedly elevated in the culture media of both OCE1 and FNE1 cells following arsenic exposure (Fig. 5A–B). Among the cytokines analyzed, IL-8 exhibited the highest concentrations, reaching 2117.1 ng/mL in OCE1 and 483.6 ng/mL in FNE1 after six weeks of exposure (Supplementary Table 2). Interestingly, IL-8 secretion exhibited distinct temporal dynamics between the two cell types. In OCE1 cells, IL-8 levels increased in a time-dependent manner, peaking at six weeks of arsenic exposure. Conversely, in FNE1 cells, IL-8 secretion displayed a time-dependent decrease, with concentrations at six weeks reduced compared to those observed at three weeks of exposure (Supplementary Table 2). These findings highlight the role of chronic arsenic exposure in creating a pro-inflammatory microenvironment with differential cytokine secretion patterns between both cultures, suggesting cell-type-specific inflammatory responses. To explore the downstream effects of NF-κB activation, we examined the expression of *p21* (CDKN1A), a cyclin-dependent kinase inhibitor and key regulator of cell cycle arrest. Chronic arsenic exposure significantly upregulated *p21* mRNA in both OCE1 and FNE1 cells at three and six weeks of exposure (Supplementary Fig. 7). Additionally, we assessed *Lamin B1*, a nuclear envelope protein essential for maintaining nuclear structure and chromatin organization. Arsenic exposure resulted in a significant reduction in *Lamin B1* mRNA expression in both cultures at the same time points (Supplementary Fig. 7), suggesting disruptions in nuclear architecture and genome organization. These findings highlight the role of NF-κB activation in modulating cell cycle regulation and nuclear structural integrity under chronic arsenic stress.

**Fig. 6.**
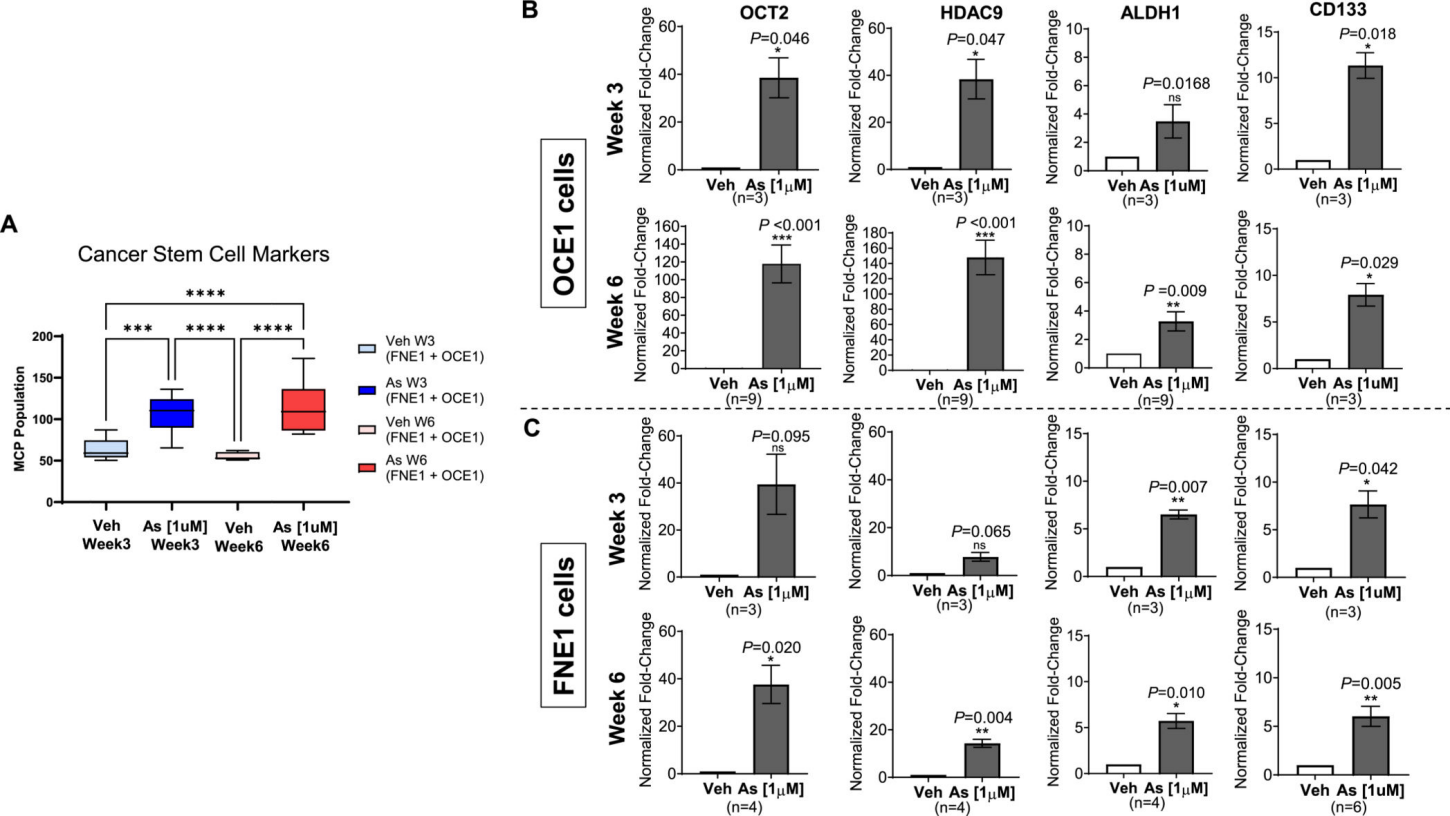
Chronic Arsenic Exposure Increases mRNA Expression Levels of Key Cancer Stem Cell Markers in OCE1 and FNE1 Cells. OCE1 and FNE1 cells were chronically exposed to 1 μM NaAsO_2_ (As) or vehicle (Veh) for 3 weeks and 6 weeks. (A) Box and whisker plot of MCP Counter population count of cells expressing cancer stem cell markers in Veh and As-exposed FNE1 and OCE1 cells. Statistical comparisons were performed using one-way ANOVA from five independent experiments for OCE1 week 3 and FNE1 weeks 3 and 6 of exposure (n = 5) and three independent experiments from OCE1 week 6 (*n* = 3), with Tukey multiple comparison correction for post-hoc comparisons. Bar graphs show the normalized fold changes in mRNA expression levels of OCT2, CD133, ALDH1, and HDAC9 in OCE1 cells at 3 weeks (B; upper panels) or 6 weeks (B; bottom panels) of exposure, and in FNE1 cells at 3 weeks (C; upper panels) or 6 weeks (C; bottom panels) of exposure. Fold changes in gene expression were determined using the delta-delta Ct method, normalized to GAPDH, and further normalized to vehicle cells from at least 3 independent experiments (n = 3–9) for each condition.

Furthermore, RNA sequencing analysis revealed increased expression of key transcription factors and markers associated with cancer stem cell (CSC) phenotypes following arsenic exposure. Using MCP counter and a list of established CSC-associated genes relevant to ovarian, cervical, and endometrial cancer stem cells (Chu et al., 2024), we identified an enrichment of CSC-like cell populations in arsenic-exposed OCE1 and FNE1 cultures (Fig. 6A). These genes included *OCT2*, *CD133*, *ALDH1*, and *HDAC9*, all of which were further validated by RT-qPCR. The analyses confirmed a significant increase in gene expression at both three and six weeks of arsenic exposure, compared to vehicle-treated controls (Fig. 6B–C). This suggests that chronic arsenic exposure may promote the early acquisition of CSC-like properties in arsenic-induced multinucleated cells. These findings collectively underscore the multifaceted role of chronic arsenic exposure in driving inflammation, genomic instability, and cellular reprogramming in ovarian surface and fallopian tube cultures.

## 4. Discussion

Arsenic represents a major global public health concern due to its widespread environmental presence in the environment and well-documented toxicity in humans (Bhat et al., 2024). Arsenic-contaminated drinking water and food crops, the primary exposure routes, affect hundreds of millions worldwide (Mandal and Suzuki, 2002). Prior epidemiological evidence links arsenic exposure with an increased risk of developing various types of cancer, including ovarian cancer (Waalkes et al., 2004; Waalkes et al., 2003). However, the precise pathways through which arsenic exerts its toxic impact on the ovary are not fully understood. Unlike other carcinogens, arsenic carcinogenicity lacks a unifying model that explains its diverse and complex effects across different tissue types, particularly in primary or human-derived cultures, which are critical for elucidating early events in arsenic-induced malignant transformation.

In this report, we investigated the cellular and molecular effects of chronic arsenic exposure using matched human normal ovarian surface and fallopian tube cultures. Employing primary heterogeneous cultures, this study provides a novel and biologically relevant *in vitro* human model to investigate arsenic-induced mechanisms of ovarian carcinogenesis. Epithelial ovarian cancer, which originates from the ovary or fallopian tube epithelium, accounts for 85–90 % of all ovarian cancer. To replicate this cellular heterogeneity and to understand the cell of origin, we used a matched set of primary cultures derived from the ovarian surface and fallopian tube epithelium of the same donor (Merritt et al., 2013). These cultures were immortalized through transfection with the pmig-GFP-hTERT vector, a method that extends cellular lifespan while preserving genomic stability (Bodnar et al., 1998). The resulting immortalized primary cultures have been well characterized and shown to resemble the genomic landscape, histopathology, and molecular features of the normal ovary (Ince et al., 2015), thereby providing a robust and relevant *in vitro* model for studying early carcinogenic events. Importantly, the observed heterogeneity in OCE1 and FNE1 primary cultures, evidenced by the presence of mixed cell populations including multiple immune cell types, highlights the utility of these models in studying the complex interplay between epithelial and immune cells under chronic arsenic exposure. The coexistence of diverse cell types within these cultures offers a unique platform to investigate the differential effects of arsenic at a cellular level, enabling the dissection of both cell-autonomous and non-cell-autonomous mechanisms of toxicity and carcinogenesis. Such heterogeneity may better represent *in vivo* conditions, where interactions between epithelial and immune cells play critical roles in shaping tissue responses to environmental stressors. Our findings demonstrated that chronic exposure to environmentally relevant concentrations of arsenic induces specific cytotoxic effects in human ovarian surface and fallopian tube cultures, which are not replicated by other toxic metals at equivalent concentrations, suggesting a unique mechanistic role for arsenic in disrupting cellular homeostasis.

A particularly intriguing observation was the morphological alteration in arsenic-exposed cultures, characterized by an enrichment of GCs and/or MNCs. These cells displayed a remarkable capacity to engulf apoptotic debris from neighboring dying cells, a phenomenon that may represent an adaptive response to arsenic-induced cytotoxic stress. The formation of these giant cells likely reflects dysregulation of key processes such as cell cycle progression, mitotic fidelity, and cytokinesis, which may be critical in enabling cell survival under chronic toxic conditions. These findings align with prior studies reporting multinucleated giant cell formation following chronic low-level arsenic exposure in immortalized human keratinocytes (Wu et al., 2012; Sun et al., 2009). Multinucleated giant cells are clinically relevant as they are associated with malignant phenotypes, metastatic potential, and chemoresistance (Lv et al., 2014; Mirzayans et al., 2018; Zhang et al., 2014). These cells contribute to tumor progression by fostering cellular heterogeneity, facilitating immune evasion, and enhancing tumor plasticity. Furthermore, their emergence following chemotherapy underscores their potential role in relapse and resistance mechanisms, highlighting their critical involvement in carcinogenesis and tumor progression.

To further elucidate the cellular origin underlying arsenic-induced morphological changes, future investigations will focus on the comprehensive phenotypic and molecular characterization of multinucleated and/or giant cells that emerge following exposure. These studies will leverage single-cell transcriptomic profiling and lineage-tracing approaches to unravel the distinct contributions of epithelial and immune cell populations. The findings of this study show that chronic arsenic exposure induces the upregulation of well-known transcription factors associated with cancer stem cell (CSC) phenotypes, including OCT2, CD133, ALDH1, and HDAC9 (Chu et al., 2024). These markers are hallmarks of stemness, self-renewal, and cellular plasticity, suggesting that arsenic-induced GCs and MNCs may acquire CSC-like properties during the process of transformation. Multinucleated giant cells, which arise under conditions of cellular stress, are increasingly recognized as critical intermediates in cancer progression, capable of generating progeny with enhanced tumorigenic potential (Zhang et al., 2014). Notably, polyploid giant cancer cells (PGCCs) have been shown to serve as a reservoir for CSCs by undergoing depolyploidization, giving rise to smaller, stem-like daughter cells that possess enhanced proliferative capacity and the ability to initiate tumor growth (Zhang et al., 2014). Similarly, Saini and colleagues demonstrated that PGCCs contribute to chemoresistance and recurrence by generating stem-like progeny, which underscores their clinical significance (Saini et al., 2022). The upregulation of OCT2, a transcription factor critical for maintaining stemness, and CD133, a surface marker of CSCs, in arsenic-induced MNCs in our study, is consistent with these prior studies and supports the hypothesis that these cells may function as precursors for CSC formation. The upregulation of HDAC9, an epigenetic modifier known to drive cellular plasticity and stem-like behavior, underscores the role of epigenetic reprogramming in arsenic-induced carcinogenesis. These molecular alterations suggest that chronic arsenic exposure not only induces polyploidization but also primes these cells for the acquisition of CSC-like traits, which may contribute to tumor initiation, progression, and resistance to therapy. However, further research is needed to elucidate the molecular mechanisms underlying the transition from multinucleated cells to CSCs and to explore therapeutic strategies targeting these cellular intermediates to prevent arsenic-induced malignancies.

Our results revealed that chronic exposure to arsenic elicits a significant increase in γ-H2AX foci in human ovarian surface and fallopian tube cultures, suggesting the accumulation of DNA double-strand breaks (DSBs). Importantly, these DSBs are not likely caused by direct genotoxicity of arsenic, but rather arise as an indirect consequence of arsenic-induced cellular stress. The observed genomic instability appears to be associated with nuclear remodeling events occurring during the formation of GC and MNCs, including nuclear fusion and aberrant cell cycle progression. Such morphological modifications may induce replication stress or disrupt chromatin architecture, thereby facilitating DSB formation. DNA DSBs represent one of the most deleterious forms of DNA lesions in cells (Khanna and Jackson, 2001) due to the simultaneous disruption of both DNA strands. Inadequate repair of DSBs can result in the loss or amplification of genomic material, chromosomal rearrangements, and the propagation of mutations, all of which are hallmarks of tumorigenesis. Our results are consistent with previous studies reporting arsenic-associated genomic instability, including DNA DSBs and DNA-protein crosslinks, in several models, including human fetal lung fibroblasts (Dong and Luo, 1993), Chinese hamster ovary cells (Ying et al., 2009), and a HeLa variant hybrid (Yih et al., 2005).

As a consequence of the chronic cellular stress induced by arsenic exposure, our results further indicate activation of the canonical DNA damage-induced NF-kB signaling pathway in human ovarian surface and fallopian tube cultures. This activation was evidenced by increased nuclear translocation of the NF-κB p65 subunit upon arsenic exposure compared to vehicle-treated controls. NF-kB is an essential transcription factor involved in several key cellular processes such as inflammation, immunity, cell proliferation, differentiation, and survival (Oeckinghaus and Ghosh, 2009). The activation of NF-κB in response to arsenic exposure triggered a robust pro-inflammatory response in both cultures, characterized by the upregulation of cytokines such as IL-1α, IL-1β, IL-6, IL-8, IL-10, and TNF-α. This sustained cytokine expression underscores the role of arsenic in promoting a pro-inflammatory microenvironment, which may contribute to the pathogenesis of ovarian cancer.

In addition to its role in inflammation, NF-κB activation influenced other critical cellular processes in response to chronic arsenic exposure. Specifically, we observed a significant increase in the expression of p21 (CDKN1A), a cyclin-dependent kinase inhibitor that plays a crucial role in cell cycle arrest. The upregulation of p21 is a hallmark of NF-κB downstream signaling and reflects a cellular attempt to mitigate DNA damage by halting proliferation. Conversely, Lamin B1, a nuclear envelope protein essential for maintaining nuclear structure and chromatin organization, was significantly decreased in arsenic-exposed ovarian surface and fallopian tube cultures. This reduction in Lamin B1 correlates with the nuclear morphological changes observed in arsenic-induced GCs and MNCs. These findings implicate the NF-κB pathway not only in the regulation of inflammatory responses but also in modulating cell cycle regulators and nuclear structural integrity, underscoring its multifaceted role in the cellular response to chronic arsenic exposure.

The NF-κB signaling pathway has been implicated in the tumorigenic effects of arsenic exposure across various models, as demonstrated in epidemiological, *in vitro*, and *in vivo* studies (Tutkun et al., 2019; Lyu et al., 2020; Yan et al., 2020; Yin and Yu, 2018; Yajima et al., 2017; Wei et al., 2016; Ahmed et al., 2011). However, our study provides the first evidence of NF-kB pathway activation and its associated pro-inflammatory response in normal human ovarian surface and fallopian tube cultures exposed to arsenic. This novel finding highlights the interplay between arsenic-associated DNA damage and inflammatory signaling pathways, revealing a potential mechanism by which arsenic exposure contributes to ovarian carcinogenesis. Future studies are needed to delineate the downstream effects of NF-κB activation, particularly its role in the progression from chronic inflammation to malignancy in ovarian surface and fallopian tube cultures.

**Fig. 7.**
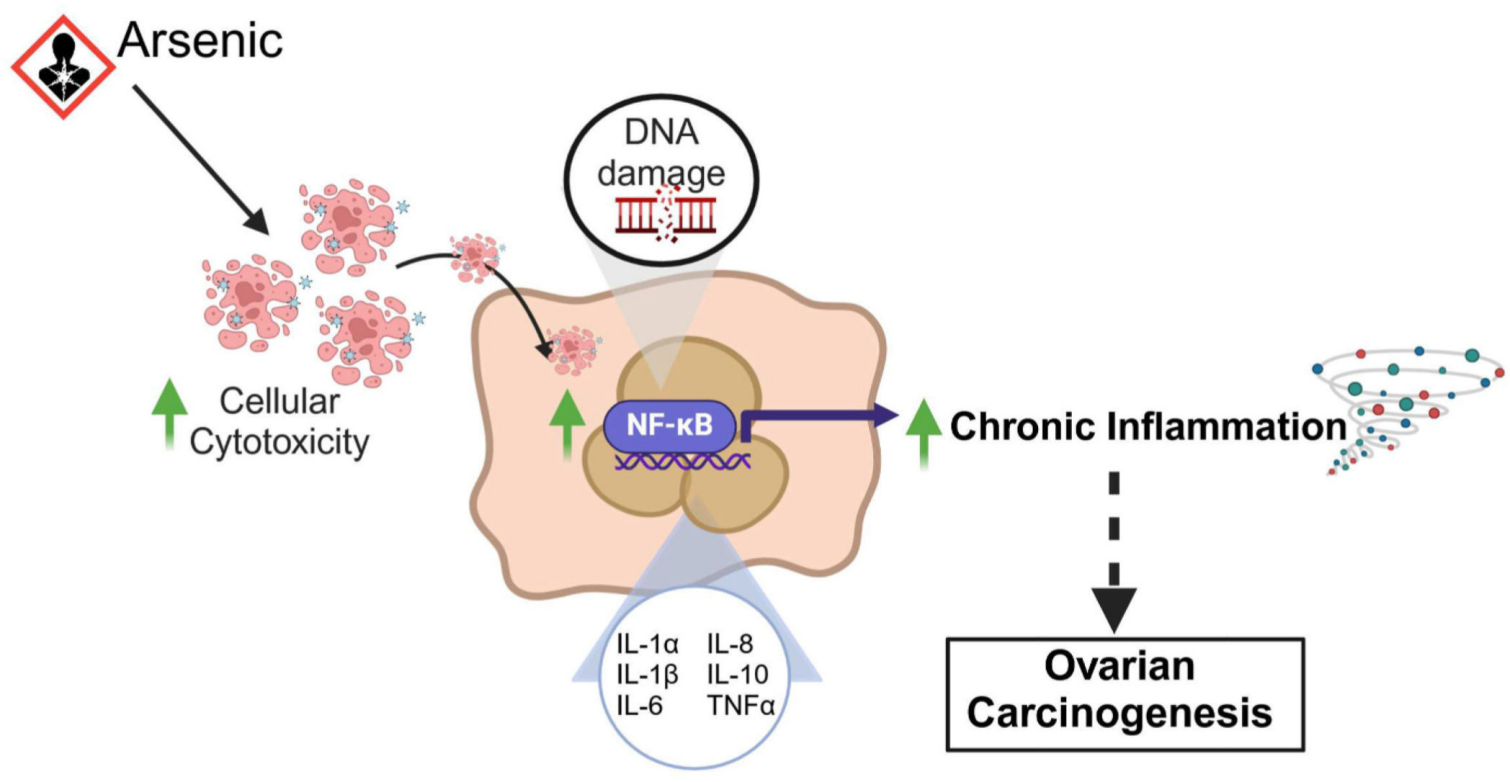
Proposed Working Model Illustrating the Impact of Chronic Arsenic Exposure on Cellular and Molecular Pathways in Ovarian Cultures. This model summarizes the cellular and molecular mechanisms underlying the effects observed in ovarian cultures chronically exposed to 1 μM NaAsO_2_. Chronic arsenic exposure induces cellular cytotoxicity and drives multinucleated and giant cell formation. These As-induced GC and MNCs display phagocytosis-like properties, actively engulfing apoptotic debris. Chronic arsenic exposure causes significant DNA damage, evidenced by the accumulation of γH2AX foci, and promotes the nuclear translocation of NF-ĸB p65, activating the canonical NF-ĸB pathway. This in turn triggers a potent inflammatory response, increasing the expression and secretion of pro-inflammatory cytokines such as TNFα, IL-6, and IL-8. Collectively, these events contribute to the disruption of cellular homeostasis and the establishment of a pro-inflammatory and potentially tumorigenic microenvironment in ovarian cultures. Image created with BioRender.com.

Together, these findings indicate that chronic arsenic exposure induces cellular cytotoxicity and drives the formation of GCs and MNCs with phagocytosis-like properties, actively engulfing apoptotic debris. Chronic arsenic exposure promotes genomic instability through indirect mechanisms such as oxidative stress, nuclear remodeling, and aberrant cell cycle progression, processes that culminate in the accumulation of γH2AX foci, a marker of DNA double-strand breaks. This genomic stress is accompanied by the nuclear translocation of NF-ĸB p65 subunit, activating the canonical NF-ĸB pathway. In turn, this activation triggers a potent inflammatory response, increasing the expression and secretion of pro-inflammatory cytokines. Collectively, these events contribute to the disruption of cellular homeostasis and the establishment of a pro-inflammatory and potentially tumorigenic microenvironment in ovarian cultures (Fig. 7).

## 5. Conclusions

Chronic exposure to environmentally relevant levels of arsenic induces marked cellular, molecular, and morphological changes in human ovarian surface and fallopian tube cultures. By leveraging a physiologically relevant *in vitro* model that captures the inherent cellular heterogeneity of the female reproductive tract, we uncovered a multifaceted toxicological response to arsenic exposure that includes nuclear remodeling, activation of the canonical NF-ĸB inflammatory pathway, and indirectly mediated DNA damage. Rather than acting as a direct genotoxin, arsenic likely promotes genomic instability through mechanisms such as oxidative stress, replication stress, and disrupted nuclear architecture, conditions that facilitate the observed accumulation of DNA double-strand breaks. The induction of GCs and MNCs, coupled with DNA double-strand breaks and NF-κB-driven pro-inflammatory cytokine expression, suggests a coordinated cellular adaptation to persistent stress that may predispose cells to malignant transformation. Importantly, our findings position GCs and MNCs as potential intermediaries in arsenic-induced ovarian carcinogenesis, capable of contributing to tumor heterogeneity, inflammation, and therapy resistance through stem-like reprogramming. Future studies aimed at elucidating the transition from chronic stress responses to oncogenic processes will be critical for understanding the role of arsenic in the pathogenesis of ovarian cancer.

## Supplementary Material

Supplementary Figure and Legends

Supplementary Video Legends

Supplementary Video 1

Supplementary Video 2

Supplementary Video 3

Supplementary Video 4

Supplementary Video 5

Supplementary Video 6

Supplementary Tables

Supplementary data to this article can be found online at https://doi.org/10.1016/j.taap.2025.117394.

## Data Availability

Data will be made available on request.

## Glossary

As: Arsenic
CSC: cancer stem cell
DEG: differentially expressed genes
FNE1: fallopian tube cultures
GCs: giant cells
MNCs: multinucleated cells
NaAsO_2_: sodium arsenite
OCE1: ovarian cultures
PBS: Phosphate Buffered Saline
PGCCs: polyploid giant cancer cells
Ppb: parts per billion
Ppm: parts per million
TBS: Tris Buffered Saline
