# Supplementary material for: Chronic arsenic exposure of ovarian surface and fallopian tube cultures induces giant and/or multinucleated cells with phagocytosis-like properties and an inflammatory phenotype
Source: Toxicol Appl Pharmacol. Author manuscript; Available in PMC 2025 Sep 8. (PMC12415987; doi:10.1016/j.taap.2025.117394)
Supplement: Supplementary Figure and Legends [file NIHMS2100229-supplement-Supplementary_Figure_and_Legends.pdf]

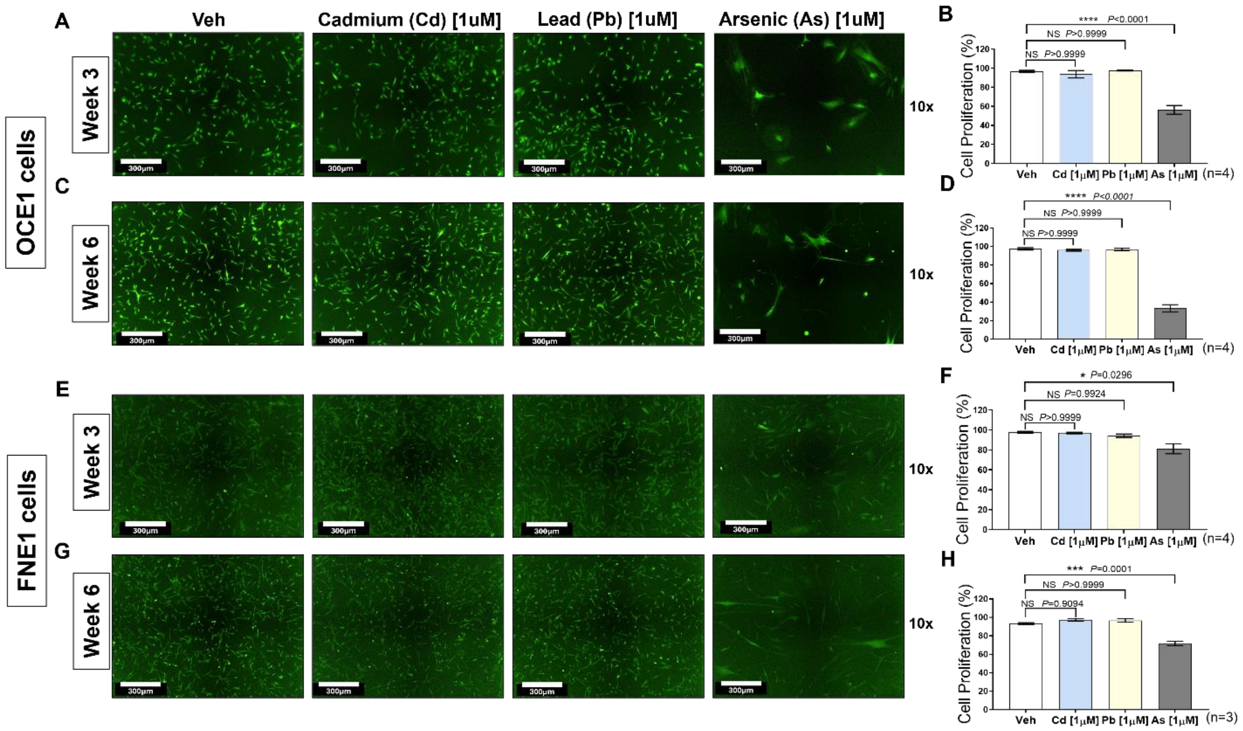

Supplementary Figure 1. Time-Dependent Arsenic-Specific Effect on Cell Proliferation in OCE1 and FNE1 Cells. OCE1 and FNE1 cells were chronically exposed to 1µM of $NaAsO_2$ (As), cadmium chloride (Cd), lead acetate (Pb), or vehicle (Veh) for 3 and 6 weeks, and monitored using the Incucyte Live Cell Imaging System. Representative live-cell images of OCE1 cells after 3 weeks (A) or 6 weeks (C) of exposure and FNE1 cells after 3 weeks (E) or 6 weeks (G) of exposure. Quantification of cellular proliferation in OCE1 cells after 3 weeks (B) or 6 weeks (D) of exposure and FNE1 cells after 3 weeks (F) or 6 weeks (H) of exposure, as determined by the total live cell area per well over time, using the Incucyte ZOOM Live Cell Analysis System. Data are presented as mean ± SEM of at least three independent experiments (n = 3-4). Statistical significance was determined using a two-tailed unpaired t-test (*$p < 0.05$, **$p < 0.01$, ***$p < 0.001$, ****$p < 0.0001$). Scale bars represent 300 µm. Images were captured at 10× magnification.

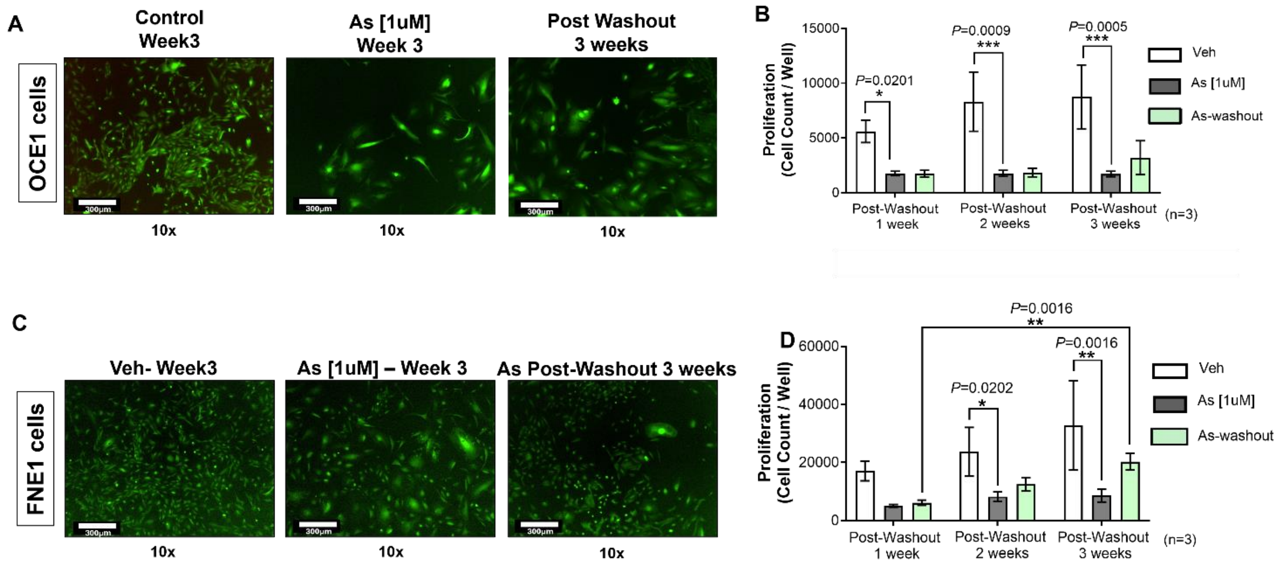

Supplementary Figure 2. Sustained Cell Proliferation in OCE1 and FNE1 Cells After Arsenic Washout. OCE1 and FNE1 cells were chronically exposed to 1µM $NaAsO_2$ (As) for 3 weeks, after which arsenic was removed from the culture media, and cells were maintained in arsenic-free media for an additional 3 weeks (Post-Washout). Live-cell imaging was performed using the Incucyte Live Cell Imaging System to assess proliferation dynamics. Representative live-cell images of OCE1 cells (A) and FNE1 cells (C) at 3 weeks of exposure to As or vehicle (Veh), and after 3 weeks of Post-Washout. Quantitative analysis of OCE1 cell proliferation (B) or FNE1 cell proliferation (D) over time, as determined by the total live cell area per well. Data are presented as mean ± SEM from three independent experiments (n = 3). Statistical significance was determined using one-way ANOVA (*p < 0.05, **p < 0.01, ***p < 0.001). Scale bars represent 300 µm, and images were captured at 10x magnification.

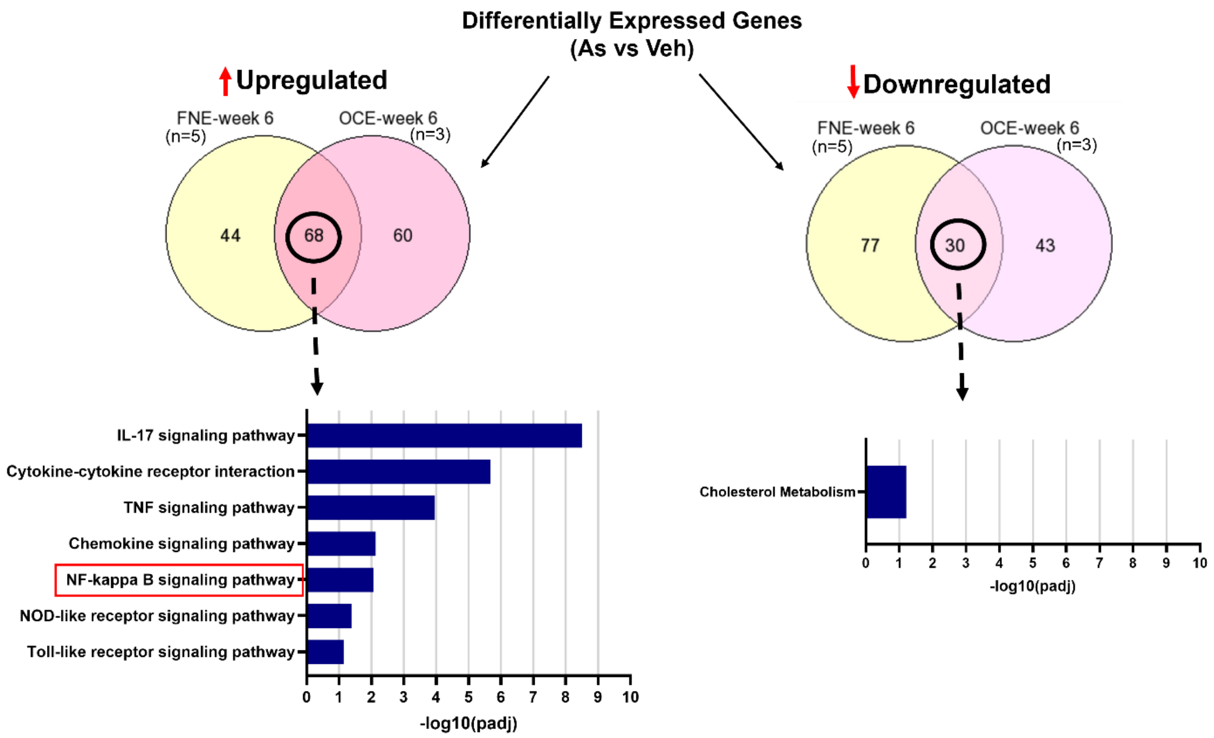

Supplementary Figure 3. RNA Sequencing Reveals Upregulated NF-kB and Inflammatory Signaling Pathways in OCE1 and FNE1 Cells Chronically Exposed to Arsenic. Venn Diagrams showing the differentially expressed genes (DEGs) commonly downregulated (30 DEGs) and upregulated (68 DEGs) in OCE1 and FNE1 cells exposed to 1μM $NaAsO_2$ (As) compared to vehicle (Veh) for 6 weeks. Bar graphs show the enrichment of downregulated and upregulated pathways of interest based on DEGs commonly expressed in OCE1 and FNE1 cells. Pathways implicated by DEGs are determined by KEGG pathway analysis using the online software DAVID (Database for Annotation, Visualization and Integrated Discovery, https://davidbioinformatics.nih.gov/). Pathways are ranked by the magnitude of significance, represented as -log10 (padj), with higher values indicating greater statistical significance. Data are presented as the mean -log10(padj) of five independent biological replicates for FNE1-week 6 of exposure (n = 5) and three independent biological replicates for OCE1-week 6 of exposure (n = 3).

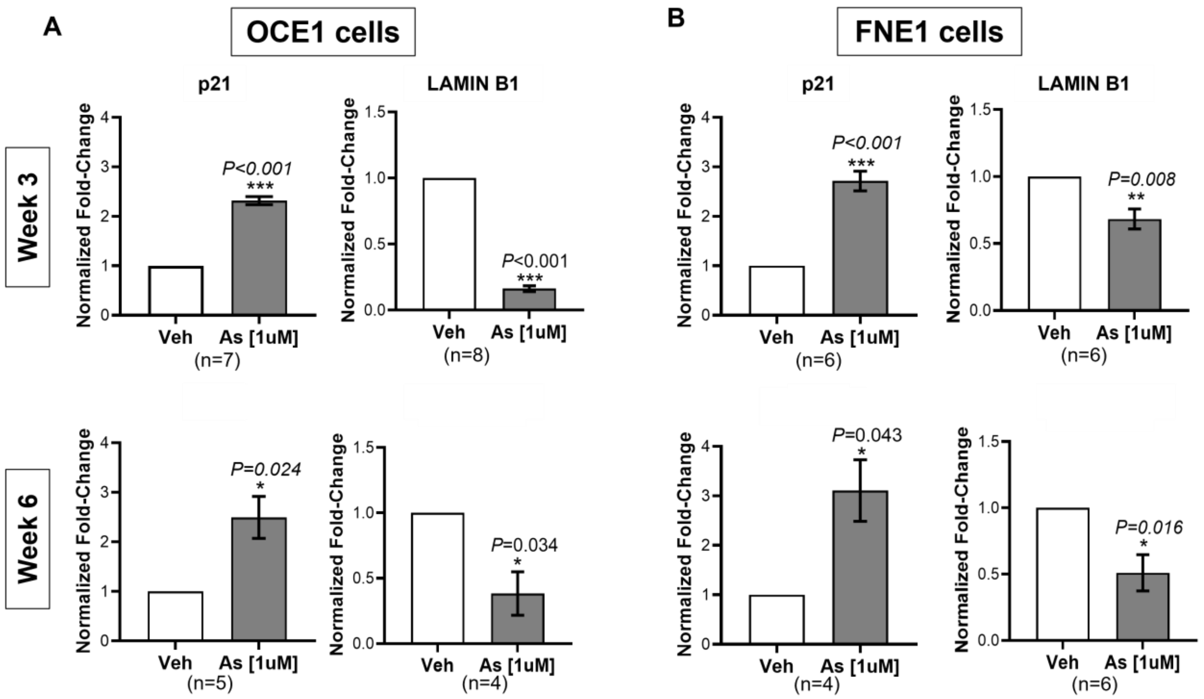

Supplementary Figure 4. Chronic Arsenic Exposure Increases mRNA Expression Levels of p21 and Decreases Lamin B1 mRNA Expression in OCE1 and FNE1 Cells. OCE1 and FNE1 cells were chronically exposed to 1μM $NaAsO_2$ (As) or vehicle (Veh) for 3 weeks and 6 weeks and mRNA expression was measured by RT-PCR. Bar graphs show the normalized fold changes in mRNA expression levels of p21 and Lamin B1 in OCE1 cells after 3 weeks (A) or 6 weeks (B) of exposure, and in FNE1 cells after 3 weeks (C) or 6 weeks (D) of exposure. Fold changes in gene expression were determined using the delta-delta Ct method, normalized to GAPDH, and further normalized to vehicle cells from at least 4 independent experiments (n=4-8) for each condition.

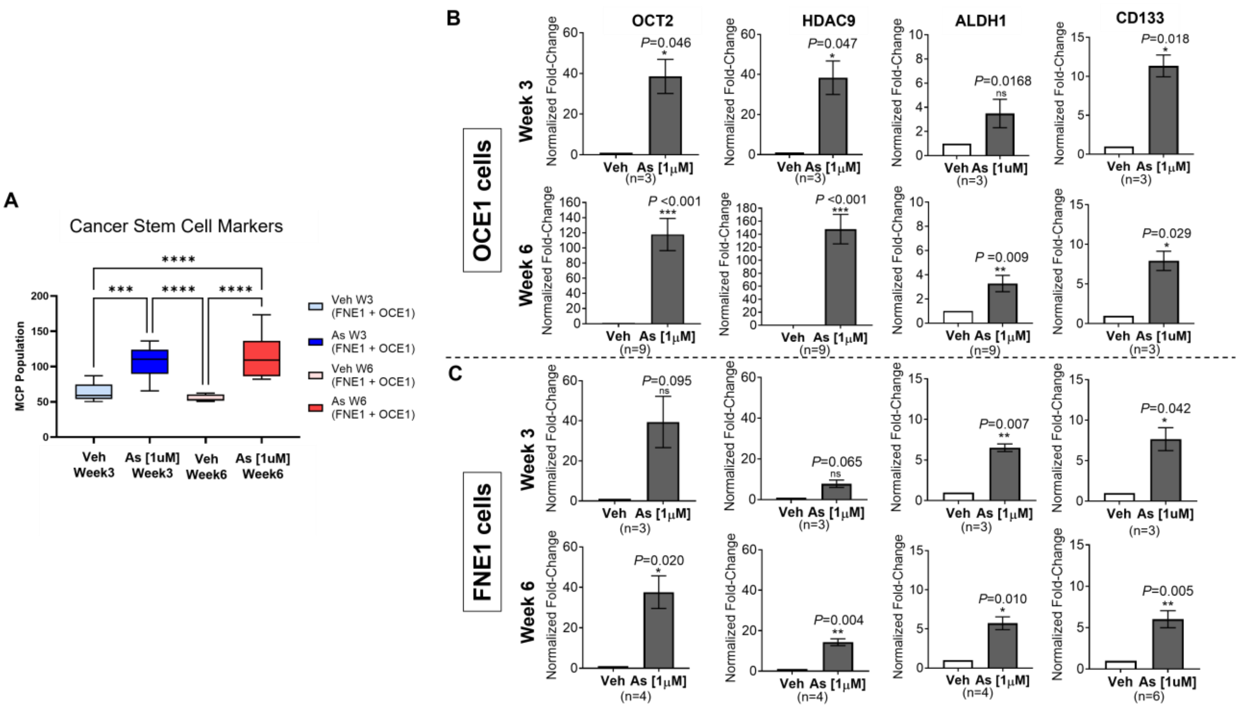

Supplementary Figure 5. Chronic Arsenic Exposure Increases mRNA Expression Levels of Key Cancer Stem Cell Transcription Factors in OCE1 and FNE1 Cells. OCE1 and FNE1 cells were chronically exposed to 1μM $NaAsO_2$ (As) or vehicle (Veh) for 3 weeks and 6 weeks. (A) Box and whisker plot of MCP Counter population count of cells expressing cancer stem cell markers in Veh and As-exposed FNE1 and OCE1 cells. Statistical comparisons were performed using one-way ANOVA from five independent experiments for OCE1 week 3 and FNE1 weeks 3 and 6 of exposure (n = 5) and three independent experiments from OCE1 week 6 (n = 3), with Tukey multiple comparison correction for post-hoc comparisons. Bar graphs show the normalized fold changes in mRNA expression levels of OCT2, CD133, ALDH1, and HDAC9 in OCE1 cells at 3 weeks (B; upper panels) or 6 weeks (B; bottom panels) of exposure, and inFNE1 cells at 3 weeks (C; upper panels) or 6 weeks (C; bottom panels) of exposure. Fold changes in gene expression were determined using the delta-delta Ct method, normalized to GAPDH, and further normalized to vehicle cells from at least 3 independent experiments (n=3-9) for each condition.

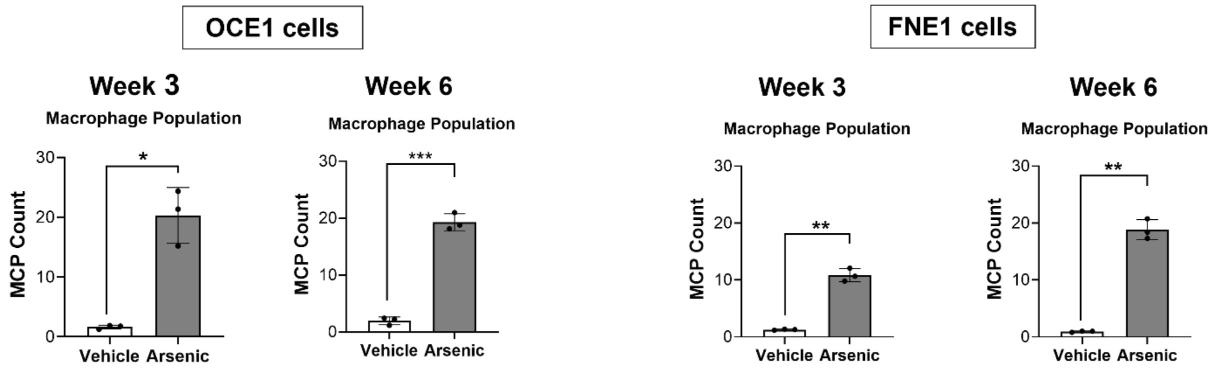

Supplementary Figure 6. Chronic Arsenic Exposure Increases Macrophage Population Counts in OCE1 and FNE1 Cells. The macrophage population within OCE1 and FNE1 cultures was quantified using MCP (macrophage-specific marker) counts obtained from RNA-seq data. Bar graphs illustrate the macrophage counts in arsenic-treated cells compared to vehicle at both 3 and 6 weeks for each OCE 1 and FNE1 cells. Data are presented as mean ± SEM from three independent experiments, and statistical significance was determined using a two-tailed unpaired t-test (*p < 0.05, **p < 0.01, ***p < 0.001).
