# Supplementary material for: Chronic arsenic exposure of ovarian surface and fallopian tube cultures induces giant and/or multinucleated cells with phagocytosis-like properties and an inflammatory phenotype
Source: Toxicol Appl Pharmacol. Author manuscript; Available in PMC 2025 Sep 8. (PMC12415987; doi:10.1016/j.taap.2025.117394)
Supplement: Supplementary Video Legends [file NIHMS2100229-supplement-Supplementary_Video_Legends.pdf]

Video Legends

Supplementary Video 1: Time-lapse imaging of OCE1 cells chronically exposed to 1 μM $NaAsO_2$ from week 3 to week 4 of exposure. The sequence begins on day 10, corresponding to 3.5 weeks of exposure, and continues until day 14, corresponding to week 4 of arsenic exposure. The white circle highlights a giant cell actively engulfing apoptotic remnants from neighboring dying cells. Images were captured at 10x magnification every 30 minutes using the Incucyte ZOOM Live Cell Analysis System.

Supplementary Video 2: Time-lapse imaging of OCE1 cells chronically exposed to vehicle from week 3 to week 4 of exposure. The sequence begins on day 10, corresponding to 3.5 weeks of exposure, and continues until day 14, corresponding to week 4 of arsenic exposure. Due to high confluency, cells were trypsinized and replated on day 13. Images were captured at 10x magnification every 30 minutes using the Incucyte ZOOM Live Cell Analysis System.

Supplementary Video 3: Time-lapse imaging of OCE1 cells chronically exposed to 1 μM $NaAsO_2$ from week 3 to week 4 of exposure. The sequence begins on day 7, corresponding to 3.5 weeks of exposure, and continues until day 13, corresponding to week 4 of arsenic exposure. The white circle highlights a giant cell actively engulfing apoptotic remnants from neighboring dying cells. Images were captured at 10x magnification every 30 minutes using the Incucyte ZOOM Live Cell Analysis System.

Supplementary Video 4: Time-lapse imaging of OCE1 cells chronically exposed to vehicle from week 3 to week 4 of exposure. The sequence begins on day 7, corresponding to 3.5 weeks of exposure, and continues until day 13, corresponding to week 4 of arsenic exposure. Images were captured at 10x magnification every 30 minutes using the Incucyte ZOOM Live Cell Analysis System.

Supplementary Video 5: Time-lapse imaging of FNE1 cells chronically exposed to 1 μM $NaAsO_2$ from week 3 to week 4 of exposure. The sequence begins on day 7, corresponding to 3.5 weeks of exposure, and continues until day 11, marking the beginning of week 4 of arsenic exposure. The white circle highlights a giant cell actively engulfing apoptotic remnants from neighboring dying cells. Images were captured at 10x magnification every 30 minutes using the Incucyte ZOOM Live Cell Analysis System.

Supplementary Video 6: Time-lapse imaging of FNE1 cells chronically exposed to vehicle from week 3 to week 4 of exposure. The sequence begins on day 7, corresponding to 3.5 weeks of exposure, and continues until day 11, marking the beginning of week 4 of arsenic exposure. Images were captured at 10x magnification every 30 minutes using the Incucyte ZOOM Live Cell Analysis System.
