# Supplementary material for: Chronic arsenic exposure of ovarian surface and fallopian tube cultures induces giant and/or multinucleated cells with phagocytosis-like properties and an inflammatory phenotype
Source: Toxicol Appl Pharmacol. Author manuscript; Available in PMC 2025 Sep 8. (PMC12415987; doi:10.1016/j.taap.2025.117394)
Supplement: Supplementary Tables [file NIHMS2100229-supplement-Supplementary_Tables.docx]

**Supplementary Table 1**: List of down-regulated and up-regulated DEGs common to both OCE1 and FNE1 following 6 weeks of arsenic exposure compared to vehicle control cells.

| **Common DEGs** | |
| --- | --- |
| **Down-Regulated Genes** | **Up-Regulated Genes** |
| [ACKR1](http://www.ncbi.nlm.nih.gov/entrez/query.fcgi?db=Gene&cmd=search&term=ACKR1) | [AC003092.1](http://www.ncbi.nlm.nih.gov/entrez/query.fcgi?db=Gene&cmd=search&term=AC003092.1) |
| [ADAMTS15](http://www.ncbi.nlm.nih.gov/entrez/query.fcgi?db=Gene&cmd=search&term=ADAMTS15) | [ADM](http://www.ncbi.nlm.nih.gov/entrez/query.fcgi?db=Gene&cmd=search&term=ADM) |
| [ADAMTSL4](http://www.ncbi.nlm.nih.gov/entrez/query.fcgi?db=Gene&cmd=search&term=ADAMTSL4) | [AMPD3](http://www.ncbi.nlm.nih.gov/entrez/query.fcgi?db=Gene&cmd=search&term=AMPD3) |
| [APOA1](http://www.ncbi.nlm.nih.gov/entrez/query.fcgi?db=Gene&cmd=search&term=APOA1) | [C10orf10](http://www.ncbi.nlm.nih.gov/entrez/query.fcgi?db=Gene&cmd=search&term=C10orf10) |
| [ARHGAP20](http://www.ncbi.nlm.nih.gov/entrez/query.fcgi?db=Gene&cmd=search&term=ARHGAP20) | [C15orf48](http://www.ncbi.nlm.nih.gov/entrez/query.fcgi?db=Gene&cmd=search&term=C15orf48) |
| [BCAM](http://www.ncbi.nlm.nih.gov/entrez/query.fcgi?db=Gene&cmd=search&term=BCAM) | [C8orf4](http://www.ncbi.nlm.nih.gov/entrez/query.fcgi?db=Gene&cmd=search&term=C8orf4) |
| [CADM3](http://www.ncbi.nlm.nih.gov/entrez/query.fcgi?db=Gene&cmd=search&term=CADM3) | [CACNA1S](http://www.ncbi.nlm.nih.gov/entrez/query.fcgi?db=Gene&cmd=search&term=CACNA1S) |
| [CD24](http://www.ncbi.nlm.nih.gov/entrez/query.fcgi?db=Gene&cmd=search&term=CD24) | [CCL20](http://www.ncbi.nlm.nih.gov/entrez/query.fcgi?db=Gene&cmd=search&term=CCL20) |
| [CDH6](http://www.ncbi.nlm.nih.gov/entrez/query.fcgi?db=Gene&cmd=search&term=CDH6) | [CD209](http://www.ncbi.nlm.nih.gov/entrez/query.fcgi?db=Gene&cmd=search&term=CD209) |
| [CNR1](http://www.ncbi.nlm.nih.gov/entrez/query.fcgi?db=Gene&cmd=search&term=CNR1) | [CD40](http://www.ncbi.nlm.nih.gov/entrez/query.fcgi?db=Gene&cmd=search&term=CD40) |
| [GPR126](http://www.ncbi.nlm.nih.gov/entrez/query.fcgi?db=Gene&cmd=search&term=GPR126) | [CD53](http://www.ncbi.nlm.nih.gov/entrez/query.fcgi?db=Gene&cmd=search&term=CD53) |
| [MATN2](http://www.ncbi.nlm.nih.gov/entrez/query.fcgi?db=Gene&cmd=search&term=MATN2) | [COL16A1](http://www.ncbi.nlm.nih.gov/entrez/query.fcgi?db=Gene&cmd=search&term=COL16A1) |
| [METTL7A](http://www.ncbi.nlm.nih.gov/entrez/query.fcgi?db=Gene&cmd=search&term=METTL7A) | [COL7A1](http://www.ncbi.nlm.nih.gov/entrez/query.fcgi?db=Gene&cmd=search&term=COL7A1) |
| [MFAP5](http://www.ncbi.nlm.nih.gov/entrez/query.fcgi?db=Gene&cmd=search&term=MFAP5) | [CSF2](http://www.ncbi.nlm.nih.gov/entrez/query.fcgi?db=Gene&cmd=search&term=CSF2) |
| [MMP28](http://www.ncbi.nlm.nih.gov/entrez/query.fcgi?db=Gene&cmd=search&term=MMP28) | [CTSS](http://www.ncbi.nlm.nih.gov/entrez/query.fcgi?db=Gene&cmd=search&term=CTSS) |
| [OXTR](http://www.ncbi.nlm.nih.gov/entrez/query.fcgi?db=Gene&cmd=search&term=OXTR) | [CXCL2](http://www.ncbi.nlm.nih.gov/entrez/query.fcgi?db=Gene&cmd=search&term=CXCL2) |
| [PCSK9](http://www.ncbi.nlm.nih.gov/entrez/query.fcgi?db=Gene&cmd=search&term=PCSK9) | [CXCL5](http://www.ncbi.nlm.nih.gov/entrez/query.fcgi?db=Gene&cmd=search&term=CXCL5) |
| [PDE3A](http://www.ncbi.nlm.nih.gov/entrez/query.fcgi?db=Gene&cmd=search&term=PDE3A) | [CXCL6](http://www.ncbi.nlm.nih.gov/entrez/query.fcgi?db=Gene&cmd=search&term=CXCL6) |
| [PKP2](http://www.ncbi.nlm.nih.gov/entrez/query.fcgi?db=Gene&cmd=search&term=PKP2) | [CXCL8](http://www.ncbi.nlm.nih.gov/entrez/query.fcgi?db=Gene&cmd=search&term=CXCL8) |
| [PLXDC2](http://www.ncbi.nlm.nih.gov/entrez/query.fcgi?db=Gene&cmd=search&term=PLXDC2) | [DCN](http://www.ncbi.nlm.nih.gov/entrez/query.fcgi?db=Gene&cmd=search&term=DCN) |
| [PRSS23](http://www.ncbi.nlm.nih.gov/entrez/query.fcgi?db=Gene&cmd=search&term=PRSS23) | [DPP4](http://www.ncbi.nlm.nih.gov/entrez/query.fcgi?db=Gene&cmd=search&term=DPP4) |
| [RBPMS2](http://www.ncbi.nlm.nih.gov/entrez/query.fcgi?db=Gene&cmd=search&term=RBPMS2) | [EHF](http://www.ncbi.nlm.nih.gov/entrez/query.fcgi?db=Gene&cmd=search&term=EHF) |
| [RTN1](http://www.ncbi.nlm.nih.gov/entrez/query.fcgi?db=Gene&cmd=search&term=RTN1) | [FMN1](http://www.ncbi.nlm.nih.gov/entrez/query.fcgi?db=Gene&cmd=search&term=FMN1) |
| [SCARA3](http://www.ncbi.nlm.nih.gov/entrez/query.fcgi?db=Gene&cmd=search&term=SCARA3) | [G0S2](http://www.ncbi.nlm.nih.gov/entrez/query.fcgi?db=Gene&cmd=search&term=G0S2) |
| [SCN9A](http://www.ncbi.nlm.nih.gov/entrez/query.fcgi?db=Gene&cmd=search&term=SCN9A) | [GBP5](http://www.ncbi.nlm.nih.gov/entrez/query.fcgi?db=Gene&cmd=search&term=GBP5) |
| [SEMA5A](http://www.ncbi.nlm.nih.gov/entrez/query.fcgi?db=Gene&cmd=search&term=SEMA5A) | [GCNT3](http://www.ncbi.nlm.nih.gov/entrez/query.fcgi?db=Gene&cmd=search&term=GCNT3) |
| [TGM1](http://www.ncbi.nlm.nih.gov/entrez/query.fcgi?db=Gene&cmd=search&term=TGM1) | [GDF15](http://www.ncbi.nlm.nih.gov/entrez/query.fcgi?db=Gene&cmd=search&term=GDF15) |
| [TLE2](http://www.ncbi.nlm.nih.gov/entrez/query.fcgi?db=Gene&cmd=search&term=TLE2) | [GS1-600G8.5](http://www.ncbi.nlm.nih.gov/entrez/query.fcgi?db=Gene&cmd=search&term=GS1-600G8.5) |
| [TP53I11](http://www.ncbi.nlm.nih.gov/entrez/query.fcgi?db=Gene&cmd=search&term=TP53I11) | [HDAC9](http://www.ncbi.nlm.nih.gov/entrez/query.fcgi?db=Gene&cmd=search&term=HDAC9) |
| [UPK1B](http://www.ncbi.nlm.nih.gov/entrez/query.fcgi?db=Gene&cmd=search&term=UPK1B) | [HKDC1](http://www.ncbi.nlm.nih.gov/entrez/query.fcgi?db=Gene&cmd=search&term=HKDC1) |
|  | [HMOX1](http://www.ncbi.nlm.nih.gov/entrez/query.fcgi?db=Gene&cmd=search&term=HMOX1) |
|  | [HSD11B1](http://www.ncbi.nlm.nih.gov/entrez/query.fcgi?db=Gene&cmd=search&term=HSD11B1) |
|  | [IGFN1](http://www.ncbi.nlm.nih.gov/entrez/query.fcgi?db=Gene&cmd=search&term=IGFN1) |
|  | [IL1B](http://www.ncbi.nlm.nih.gov/entrez/query.fcgi?db=Gene&cmd=search&term=IL1B) |
|  | [IL24](http://www.ncbi.nlm.nih.gov/entrez/query.fcgi?db=Gene&cmd=search&term=IL24) |
|  | [IL4I1](http://www.ncbi.nlm.nih.gov/entrez/query.fcgi?db=Gene&cmd=search&term=IL4I1) |
|  | [IQCD](http://www.ncbi.nlm.nih.gov/entrez/query.fcgi?db=Gene&cmd=search&term=IQCD) |
|  | [IRAK2](http://www.ncbi.nlm.nih.gov/entrez/query.fcgi?db=Gene&cmd=search&term=IRAK2) |
|  | [IVL](http://www.ncbi.nlm.nih.gov/entrez/query.fcgi?db=Gene&cmd=search&term=IVL) |
|  | [KCNJ15](http://www.ncbi.nlm.nih.gov/entrez/query.fcgi?db=Gene&cmd=search&term=KCNJ15) |
|  | [KYNU](http://www.ncbi.nlm.nih.gov/entrez/query.fcgi?db=Gene&cmd=search&term=KYNU) |
|  | [LCN2](http://www.ncbi.nlm.nih.gov/entrez/query.fcgi?db=Gene&cmd=search&term=LCN2) |
|  | [LINC00504](http://www.ncbi.nlm.nih.gov/entrez/query.fcgi?db=Gene&cmd=search&term=LINC00504) |
|  | [LINC00520](http://www.ncbi.nlm.nih.gov/entrez/query.fcgi?db=Gene&cmd=search&term=LINC00520) |
|  | [NRROS](http://www.ncbi.nlm.nih.gov/entrez/query.fcgi?db=Gene&cmd=search&term=NRROS) |
|  | [PI3](http://www.ncbi.nlm.nih.gov/entrez/query.fcgi?db=Gene&cmd=search&term=PI3) |
|  | [PILRA](http://www.ncbi.nlm.nih.gov/entrez/query.fcgi?db=Gene&cmd=search&term=PILRA) |
|  | [PIP](http://www.ncbi.nlm.nih.gov/entrez/query.fcgi?db=Gene&cmd=search&term=PIP) |
|  | [PLA2G4C](http://www.ncbi.nlm.nih.gov/entrez/query.fcgi?db=Gene&cmd=search&term=PLA2G4C) |
|  | [POSTN](http://www.ncbi.nlm.nih.gov/entrez/query.fcgi?db=Gene&cmd=search&term=POSTN) |
|  | [POU2F2](http://www.ncbi.nlm.nih.gov/entrez/query.fcgi?db=Gene&cmd=search&term=POU2F2) |
|  | [QPCT](http://www.ncbi.nlm.nih.gov/entrez/query.fcgi?db=Gene&cmd=search&term=QPCT) |
|  | [RP13-143G15.4](http://www.ncbi.nlm.nih.gov/entrez/query.fcgi?db=Gene&cmd=search&term=RP13-143G15.4) |
|  | [RPLP0P2](http://www.ncbi.nlm.nih.gov/entrez/query.fcgi?db=Gene&cmd=search&term=RPLP0P2) |
|  | [S100A9](http://www.ncbi.nlm.nih.gov/entrez/query.fcgi?db=Gene&cmd=search&term=S100A9) |
|  | [SAA1](http://www.ncbi.nlm.nih.gov/entrez/query.fcgi?db=Gene&cmd=search&term=SAA1) |
|  | [SAA2](http://www.ncbi.nlm.nih.gov/entrez/query.fcgi?db=Gene&cmd=search&term=SAA2) |
|  | [SAA4](http://www.ncbi.nlm.nih.gov/entrez/query.fcgi?db=Gene&cmd=search&term=SAA4) |
|  | [SERPINB7](http://www.ncbi.nlm.nih.gov/entrez/query.fcgi?db=Gene&cmd=search&term=SERPINB7) |
|  | [SIRPB1](http://www.ncbi.nlm.nih.gov/entrez/query.fcgi?db=Gene&cmd=search&term=SIRPB1) |
|  | [SLAMF8](http://www.ncbi.nlm.nih.gov/entrez/query.fcgi?db=Gene&cmd=search&term=SLAMF8) |
|  | [SLC16A6](http://www.ncbi.nlm.nih.gov/entrez/query.fcgi?db=Gene&cmd=search&term=SLC16A6) |
|  | [SLC43A3](http://www.ncbi.nlm.nih.gov/entrez/query.fcgi?db=Gene&cmd=search&term=SLC43A3) |
|  | [STAT4](http://www.ncbi.nlm.nih.gov/entrez/query.fcgi?db=Gene&cmd=search&term=STAT4) |
|  | [TNFAIP6](http://www.ncbi.nlm.nih.gov/entrez/query.fcgi?db=Gene&cmd=search&term=TNFAIP6) |
|  | [VNN3](http://www.ncbi.nlm.nih.gov/entrez/query.fcgi?db=Gene&cmd=search&term=VNN3) |
|  | [ZC3H12A](http://www.ncbi.nlm.nih.gov/entrez/query.fcgi?db=Gene&cmd=search&term=ZC3H12A) |
|  | [ZNF469](http://www.ncbi.nlm.nih.gov/entrez/query.fcgi?db=Gene&cmd=search&term=ZNF469) |

**Supplementary Table 2:** Protein average concentration (pg/mL) of each pro-inflammatory cytokine (IL1α, IL1β, IL6, IL8, IL10, and TNF-α) in both OCE1 and FNE1 cells after 3 weeks and 6 weeks of exposure to Arsenic or Vehicle.

| **Cells** | **Week** | **Cytokine** | **Dilution** | **Average Concentration (pg/mL) (N=5)** | | **p-value** | ***** |
| --- | --- | --- | --- | --- | --- | --- | --- |
|  |  |  |  | **Vehicle** | **Arsenic** |  |  |
| OCE1 | Week 3 | IL10 | [1:10] | 0.8626 | 5.308 | p=0.028 | * |
|  |  | IL1a | [no dilution] | 4.311 | 59.16 | p=0.042 | * |
|  |  | IL1b | [1:10] | 4.937 | 35.41 | p<0.001 | *** |
|  |  | IL6 | [1:10] | 134.4 | 3815 | p=0.021 | * |
|  |  | IL8 | [1:8,000] | 46940 | 1104152 | p=0.017 | * |
|  |  | TNFa | [1:10] | 3.629 | 18.52 | p=0.031 | * |
|  | Week 6 | IL10 | [1:10] | 0.9507 | 4.507 | p<0.001 | *** |
|  |  | IL1a | [no dilution] | 5.533 | 197.7 | p=0.047 | * |
|  |  | IL1b | [1:10] | 3.669 | 35.81 | p=0.001 | ** |
|  |  | IL6 | [1:10] | 131.9 | 4894 | p=0.001 | ** |
|  |  | IL8 | [1:8,000] | 86759 | 2117103 | p=0.019 | * |
|  |  | TNFa | [1:10] | 3.85 | 20.65 | p<0.001 | *** |
| FNE1 | Week 3 | IL10 | [1:10] | 0.8412 | 6.326 | p<0.001 | *** |
|  |  | IL1a | [no dilution] | 9.815 | 161.5 | p=0.008 | ** |
|  |  | IL1b | [1:10] | 0.5181 | 40.23 | p<0.001 | *** |
|  |  | IL6 | [1:10] | 1.966 | 4302 | p<0.001 | *** |
|  |  | IL8 | [1:8,000] | 43692 | 2339608 | p=0.012 | * |
|  |  | TNFa | [1:10] | 1.644 | 20.31 | p<0.001 | *** |
|  | Week 6 | IL10 | [1:10] | 0.4961 | 2.094 | p<0.001 | *** |
|  |  | IL1a | [no dilution] | 7.201 | 84.42 | p=0.049 | * |
|  |  | IL1b | [1:10] | 2.569 | 10.78 | p<0.001 | *** |
|  |  | IL6 | [1:10] | 12.34 | 545.9 | p=0.029 | * |
|  |  | IL8 | [1:8,000] | 105229 | 483594 | p=0.022 | * |
|  |  | TNFa | [1:10] | 1.922 | 8.185 | p=0.014 | * |
